# Effect of Competing Metals and Humic Substances on Uranium Mobilization from Noncrystalline U(IV) Induced by Anthropogenic and Biogenic Ligands

**DOI:** 10.1021/acs.est.3c01705

**Published:** 2023-10-11

**Authors:** Kyle J. Chardi, Walter D. C. Schenkeveld, Naresh Kumar, Daniel E. Giammar, Stephan M. Kraemer

**Affiliations:** † Centre for Microbiology and Environmental Systems Science, Department for Environmental Geosciences, University of Vienna, Josef-Holaubek-Platz 2 1090 Vienna, Austria; ‡ Soil Chemistry and Chemical Soil Quality Group, Wageningen University and Research, Droevendaalsesteeg 3, 6708 PB Wageningen, The Netherlands; § Department of Energy, Environmental, and Chemical Engineering, One Brookings Drive, Washington University, St. Louis, Missouri 63130, United States

**Keywords:** uranium, chelating ligands, competing metals, humic substances, exchange reactions

## Abstract

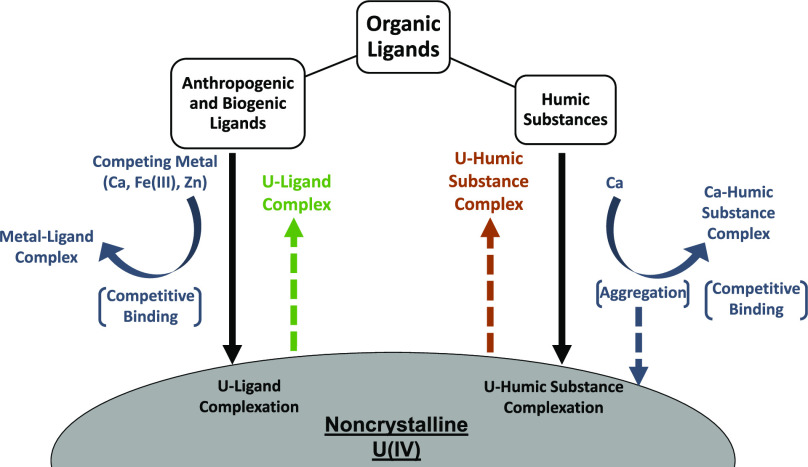

Anthropogenic and biogenic ligands may mobilize uranium (U) from tetravalent U (U(IV)) phases in the subsurface, especially from labile noncrystalline U(IV). The rate and extent of U(IV) mobilization are affected by geochemical processes. Competing metals and humic substances may play a decisive role in U mobilization by anthropogenic and biogenic ligands. A structurally diverse set of anthropogenic and biogenic ligands was selected for assessing the effect of the aforementioned processes on U mobilization from noncrystalline U(IV), including 2,6-pyridinedicarboxylic acid (DPA), citrate, *N*,*N*′-di(2-hydroxybenzyl)ethylene-diamine-*N*,*N*′-diacetic acid (HBED), and desferrioxamine B (DFOB). All experiments were performed under anoxic conditions at pH 7.0. The effect of competing metals (Ca, Fe(III), and Zn) on ligand-induced U mobilization depended on the particular metal–ligand combination ranging from nearly complete U mobilization inhibition (e.g., Ca-citrate) to no apparent inhibitory effects or acceleration of U mobilization (e.g., Fe(III)-citrate). Humic substances (Suwannee River humic acid and fulvic acid) were tested across a range of concentrations either separately or combined with the aforementioned ligands. Humic substances alone mobilized appreciable U and also enhanced U mobilization in the presence of anthropogenic or biogenic ligands. These findings illustrate the complex influence of competing metals and humic substances on U mobilization by anthropogenic and biogenic ligands in the environment.

## Abstract 2

Other metals compete with U for complexation by chelating ligands, hindering ligand-induced U mobilization, while humic substances contribute to ligand-induced U mobilization from noncrystalline U(IV) under reducing conditions.

## Introduction

The mobility of uranium (U) in the environment is predominantly controlled by its redox state with hexavalent U (U(VI)) being highly soluble and tetravalent U (U(IV)) being sparingly soluble.^1^ The limited solubility of U(IV) is utilized in bioremediation efforts to diminish dissolved U concentrations in the subsurface by the addition of an organic electron donor.^2−4^ U(VI) bioreduction processes can yield both relatively stable crystalline phases, such as uraninite (UO_2_), and more labile phases, such as noncrystalline U(IV).^5−8^ Noncrystalline U(IV) is defined as U(IV) species for which the extended X-ray absorption fine structure (EXAFS) Fourier transform spectra lack U–U pair correlations, a characteristic of crystalline U(IV) phases.^9^ Noncrystalline U(IV) commonly binds to phosphate or carboxyl groups of biomass^10^ and is a predominant end product of U bioremediation efforts and commonly reported in natural settings, such as roll front formations.^6,8,9^

The groundwater matrix and local subsurface environment can significantly affect the stability of immobilized U(IV) species, especially of labile phases, such as noncrystalline U(IV). Previous studies have reported the comparatively large susceptibility of noncrystalline U(IV) to remobilization by bicarbonate and reoxidation by dissolved oxygen or persulfate compared to UO_2_.^11−13^ For example, Cerrato et al. showed that after 6 h of 50 mM K_2_S_2_O_8_ amendment, only 35% of chemogenic UO_2_ was extracted, while 90% was extracted from noncrystalline U(IV).^13^ Various metals commonly found in groundwater can additionally affect the dissolution rate of U(IV) phases through various mechanisms (e.g., surface shielding, competitive binding, etc.). For example, Cerrato et al. showed the inhibitory effects of Ca and Zn on the oxidative dissolution of UO_2_ by formation of passivating phases on the surface (Ca–U(VI) and Zn carbonate, respectively).^14^

Another concern for decreasing U mobility in the subsurface is the occurrence of organic ligands. In our previous work, we demonstrated the ability of several chelating ligands to mobilize reduced U(IV) phases and the heightened effect on noncrystalline U(IV) compared to UO_2_.^15^ These findings, in conjunction with results from other studies, bolster the concern that organic ligands may enhance the mobilization of U(IV) species in the environment.^12,16,17^

Humic substances (HS) are ubiquitous in nature, commonly associated with minerals, and play important roles in biogeochemical processes in the subsurface.^18−20^ Notably, HS have been shown to mobilize various metals and enhance metal mobilization by other organic ligands.^21,22^ Luo et al. showed the ability of both humic acid (HA) and fulvic acid (FA) to mobilize U from a bioreduced field sediment.^23^ Previous studies have confirmed the ability of HA and FA to mobilize both U(IV) and U(VI), with HA having a higher affinity for U(IV) and FA having a higher affinity for U(VI).^23−25^ Additionally, negatively charged HS covering mineral surfaces can enhance ligand-controlled dissolution by positively charged biogenic ligands such as desferrioxamine B (DFOB).^21^ HS and reducing conditions required for U(VI) reduction overlap in several environments, such as peat soils and wetlands.^26^ We therefore predict that HS could be relevant compounds for U(IV) mobilization in the environment.

Competitive complexation of other metals in the subsurface by chelating ligands has the potential to limit the rate and extent of mobilization of a specific (target) metal,^27,28^ including U. The impact of such competitive reactions depends on various factors, including soil properties, ligand concentration, and characteristics of the ligand such as denticity, type of functional groups, and molecular-scale structure.^29,30^ A number of studies have shown the ability of competing metals (e.g., calcium, zinc) to significantly hinder complexation of a target metal by organic ligands (including low-molecular-weight organic acids, synthetic chelators, and siderophores) through competitive binding.^28,31,32^ Furthermore, HS, especially HA, can coagulate when complexing multivalent metals such as calcium, decreasing their ability to form soluble complexes with metals such as U.^33^

The objective of the current work was to provide a detailed understanding of processes that govern ligand-induced U mobilization from noncrystalline U(IV) species under anoxic conditions in the environment. Notably, we refer to DPA, citrate, HBED, and DFOB as “anthropogenic and biogenic ligands” or, in short, “ligands” in the remainder of the manuscript for simplicity. Similarly, we refer to Suwannee River humic acid and Suwannee River fulvic acid as “humic substances”, being well aware that HS act as ligands as well. In the context of our objective, we carried out a series of controlled laboratory batch experiments. First, the role of metal competition in ligand-induced U mobilization was probed with several metals commonly occurring in soils and sediments. The selected metals were calcium (Ca, a dominant exchangeable cation in temperate soils, abundant in calcareous soils: >10% calcium content), ferric iron (Fe(III), the fourth most abundant element in the earth’s crust, soils typically comprise ∼2% iron), and zinc (Zn, an essential micronutrient, average soil concentration ∼50 mg kg^–1^).^34−36^ Second, the ability of HS to mobilize U from noncrystalline U(IV) was tested across a range of HA and FA concentrations. Finally, the effect of HS pre-equilibrated with noncrystalline U(IV) on U mobilization by anthropogenic and biogenic ligands was tested. Our results illustrate the importance of accounting for metal competition reactions and the contribution of HS in assessing ligand-induced U mobilization in reducing environments.

## Materials and Methods

### Noncrystalline U(IV) Synthesis and Characterization

Noncrystalline U(IV) was synthesized as described in Bernier-Latmani et al.^9^ Briefly, U(VI) was reduced by the addition of *Shewanella oneidensis* MR-1 cultures and lactate as the organic electron donor in a Widdel low-phosphate (WLP) medium (Table S1). After 14 days of reduction, serum bottles in triplicate were sampled under anaerobic conditions and dissolved U concentrations were measured to confirm U reduction (>99.99% U removed from the solution).

After 50 mM bicarbonate rinsing, the noncrystalline U(IV) was characterized by X-ray absorption spectroscopy (XAS) analysis at beamline 4-1 of the Stanford Synchrotron Radiation Lightsource (SSRL). Linear combination fitting results confirmed that the sample predominantly comprised U(IV) (98% U(IV), 2% U(VI); no U(V) reference included in fitting results) and corresponded well with the noncrystalline U(IV) reference spectra (90% noncrystalline U(IV), 10% biogenic nanoparticulate UO_2_). Comparable biogenic UO_2_ contents in noncrystalline U(IV) samples have been reported previously.^11^ Possible residual U(VI), if any, would therefore be present at a very low concentration, as the added reference spectra did not improve the fit significantly. Ion exchange chromatography was utilized to probe the extent of U(VI) mobilized in experiments (see below). Aliquots from the noncrystalline U(IV) stock suspension were taken in duplicate and digested in 10% HNO_3_ at 100 °C for 4 h before analysis by inductively coupled plasma mass spectrometry (ICP-MS, Agilent) to confirm total U concentrations (within 1 μM of aimed U stock suspension concentration of 300 μM) prior to the start of the experiment. Further details on the noncrystalline U(IV) synthesis and characterization are provided in Chardi et al. (specifically in Text S3).^15^

### Ligands and Other Chemicals

The anthropogenic and biogenic ligands used in this study included two tridentate low-molecular-weight organic acids (2,6-pyridinedicarboxylic acid (DPA) and citrate) and two hexadentate ligands (the synthetic chelator *N*,*N*′-di(2-hydroxybenzyl)ethylene-diamine-*N*,*N*′-diacetic acid (HBED) and the microbial siderophore DFOB). The selected ligands encompass those with harder Lewis base groups (HBED and DFOB having phenolate and hydroxamate groups, respectively), expected to have a greater affinity for the hard Lewis acid U(IV), and those of softer character (citrate and DPA), expected to have a greater affinity for the relatively softer Lewis acid U(VI). These ligands are present in the environment and originate from both anthropogenic sources (e.g., fertilizers) and natural sources (e.g., plant and microbial exudates).^37−39^

Suwannee River humic acid (standard III) and Suwannee River fulvic acid (standard III) were purchased from the International Humic Substance Society (IHSS). The carbon contents of the HA and FA were 54.6 and 53.3% (w w^–1^), respectively.^40^ Anoxic deionized water (DI water, resistivity >18.2 MΩ·cm, TOC < 2 ppb, Milli-Q, Millipore) was used for all solutions and suspensions. Anoxic DI water was prepared by boiling DI water followed by N_2(g)_ purging (>3 h) while cooling down prior to introduction into the anaerobic chamber to equilibrate overnight before use. All chemicals were purchased from commercial sources, of analytical grade, and used as received.

### U Mobilization from Noncrystalline U(IV)

The effect of anthropogenic and biogenic ligands on the kinetics and extent of U mobilization from noncrystalline U(IV) (300 μM U suspension) was investigated in batch experiments. All experiments were carried out in an anaerobic (O_2_ conc. < 1 ppmv) chamber (Braun Unilab Pro) under a N_2(g)_ atmosphere for 2 days (unless stated otherwise). Experiments were performed in duplicate in 30 mL glass reactors with continuous stirring of the suspensions (nonsterile). Results were plotted as the mean (*n* = 2), with the ends of the error bars representing the measured values.

Prior to each experiment, a 50 mM anoxic bicarbonate extraction step was carried out on the noncrystalline U(IV) stock suspension for 12 h to remove residual U(VI).^11^ The suspension was centrifuged at 7000 relative centrifugal force (RCF) in Nalgene Oak Ridge centrifuge tubes (polypropylene) with sealing caps for 10 min followed by four anoxic water rinsing steps before preparing a 100 times concentrated stock suspension (30 mM U).

The pH of solutions was buffered to 7.0 using 10 mM 3-(*N*-morpholino) propanesulfonic acid (MOPS, p*K*_a_ = 7.28) unless mentioned otherwise. MOPS was selected because it does not affect U complexation, as observed in previous studies.^13,15,16^ The ionic strength was fixed to 0.01 M by the addition of NaCl unless stated otherwise (see below).

Samples were taken over time and filtered through 0.2 μm cellulose acetate filters (Sartorius) before acidification with trace-metal-grade HNO_3_ for analysis by ICP-MS. An ion exchange chromatography method was utilized to examine the redox speciation of the dissolved uranium (see below).^41,42^ Ligand-only treatments were first assessed (in the absence of competing metals and HS) for all four ligands as a reference for further experiments. Tested ligand concentrations were 0.5, 5, and 50 μM in addition to a ligand-free control.

#### Competing Metal Effects

Three metals prevalent in the subsurface (Ca, Fe(III), and Zn) were tested for their influence on ligand-induced U mobilization from noncrystalline U(IV) only at a ligand concentration of 50 μM. Metals were applied to ligand stock solutions prior to the addition of noncrystalline U(IV), allowing for precomplexation (12 h). Precomplexation was performed differently depending on the metal (Table S2). In the case of Ca, CaCl_2_ replaced NaCl as the electrolyte and was added at the proper concentration to maintain an ionic strength of 10 mM (final concentration of 2.2 mM Ca) to represent elevated calcium concentrations in the subsurface.^43,44^ The FeCl_3_ stock was first prepared at pH 2.0 to prevent Fe(hydr)oxide precipitation. Fe and ligand stocks were subsequently mixed with a small excess of Fe (2% on a molar basis). The pH was raised to 7.5 to facilitate complexation and to allow noncomplexed Fe(III) to precipitate out. Fe-ligand complex solutions were foil-wrapped and allowed to equilibrate for 12 h before filtration (0.1 μm polyvinylidene difluoride, PVDF) to remove precipitated Fe(III) (e.g., nanoparticulate ferrihydrite). Finally, solutions were prepared to a ligand concentration of 50 μM. Dissolved Fe concentrations remained >48 μM for all ligands except DPA, for which it was significantly lower: <5 μM (see the Results and Discussion Section). Zinc–ligand complex solutions were prepared by mixing dissolved ZnCl_2_ at a 1:1 stoichiometry with the ligands at 50 μM. The fraction of the ligand complexing Zn predicted by PHREEQC varied per ligand (ZnHBED^2–^, 98%; Zn-citrate^–^, 81%; ZnDPA, 44%; ZnH_2_HDFOB^+^, 16%). The model contained the ligand, Zn, and electrolyte. The Minteqv4 database was used in combination with stability constants summarized in Table S3 (detailed approach below). All Zn-containing solid phases were undersaturated in all model runs.

#### Humic Substance Effects

U mobilization from noncrystalline U(IV) by HS was probed over a range of HA and FA concentrations. Experiments were carried out at 9, 34, 60, and 85 mg C L^–1^ for HA and 9, 36, 62, and 89 mg C L^–1^ for FA, based on typical environmental HS concentrations ranging from <1 to 50 mg C L^–1^ with concentrations >100 mg C L^–1^ reported in peat bogs and wetlands.^45−47^ For stock solutions and selected treatments where MOPS was omitted, dissolved organic carbon (DOC) concentrations were measured (nonpurgeable organic carbon (NPOC); TOC-L, Shimadzu) after filtration through 0.45 μm filters (nylon, Yeti). Nylon filters were used to minimize DOC contributions from the filter matrix (as tested in a filter test, data not shown). For treatments where MOPS was omitted, the pH was closely monitored and the pH was found to deviate by only ±0.3 pH units. U mobilization in the presence and absence of MOPS buffer (0.2 μm cellulose acetate filter) resulted in comparable concentrations (U concentrations differed by <1.5 μM; data not shown). The ionic strength was set to 10 mM with either NaCl or CaCl_2_. HS aggregation and subsequent precipitation were assessed by determining DOC concentrations in selected treatments (see below).

The effect of Ca complexation by HS on U mobilization from noncrystalline U(IV) was tested using 60 mg C L^–1^ HA or 62 mg C L^–1^ FA. Also, the combined effect of 50 μM DPA added 1 day after the aforementioned HA or FA concentrations was tested. All Ca complexation experiments were carried out as detailed above (NaCl replaced as the electrolyte with CaCl_2_, equaling 2.2 mM Ca). The effects of Fe(III) and Zn on HS-induced U mobilization were not investigated due to their relatively lower concentrations in the environment.

The effect of pre-equilibration of noncrystalline U(IV) with HS on U mobilization by anthropogenic and biogenic ligands was investigated in two setups: first, noncrystalline U(IV) suspensions were pre-equilibrated with the buffer, electrolyte, and 9 mg C L^–1^ of either HA or FA for 1 day prior to ligand addition. Stocks of the various ligands were spiked into the experimental suspensions to a final concentration of 50 μM. Second, the effect of the concentration of pre-equilibrated HS on U mobilization was probed with DPA (as a model ligand). HS concentrations were varied over the same concentrations as in the HS-only treatments. Table S3 summarizes all of the experimental treatments.

### Ion Exchange Chromatography

Ion exchange chromatography was used to determine the dissolved U(IV) and U(VI) concentrations at the end of experiments (2 d) for selected treatments in accordance with Stoliker et al. and Wang et al. and is detailed in Text S1.^41,42^ Briefly, U(VI) and U(IV) were selectively eluted from filtered samples in chromatography columns loaded with anion exchange resin by either 10 pore volumes of 0.1 or 4.5 M HCl, respectively. The percent recovery of dissolved U of the U(IV) and U(VI) fractions combined was always found to be within 12% of that of the total dissolved U concentration measured on ICP-MS. The largest errors appeared at concentrations below 0.5 μM.

### Aqueous Speciation Calculations

Aqueous speciation modeling was performed using PHREEQC to predict the equilibrium speciation of each respective organic ligand (DPA, citrate, HBED, DFOB) with the corresponding concentration of the competing metals applied (Ca, Fe(III), Zn).^48^ Notably, models were run in the absence of U due to a lack of stability constants. In the case of citrate, where stability constants are available for both U(IV) and competing metals of interest, additional simulations were carried out by comparing the equilibrium speciation when both metals were added. Further details are provided in Text S2. All relevant constants used for the models are summarized in Table S4, with all models being run using the Minteqv4 database.

## Results and Discussion

### Ligand-Induced U Mobilization from Noncrystalline U(IV)

Several ligand concentrations (0.5, 5, and 50 μM) were tested for their effect on U mobilization from noncrystalline U(IV). During the experiments, the U redox state in solution was measured by ion exchange chromatography (Figure S1). These results serve as a baseline for comparison with more complex experiments, where combined effects of ligands, metals, and HS were investigated.

Results from ion exchange chromatography showed differing proportions of U(IV) and U(VI) in solution for the different ligands. Minor deviations were also observed across concentrations from the same ligand. Mobilized U in the ligand-free control (<0.1% of total U in suspension) comprised 62% U(IV) and 38% U(VI) (U(VI) accounting for <0.15 μM U). The predominance of U(IV) dissolution confirms that even in ligand-free treatments residual traces of U(VI) bound to the noncrystalline U(IV) do not primarily control U solubility. HBED, DFOB, and citrate all mobilized U, resulting in similar proportions of U(IV) and U(VI) for each ligand, equaling ∼86% U(IV) and ∼14% U(VI) of the dissolved U in solution (deviation between ligands = 0.8%). The mobilized U(VI) concentrations in these treatments (<1.8 μM U) remained below the total U(VI) concentration in suspension (∼6 μM U). This residual U(VI) presumably persisted after 50 mM bicarbonate rinsing prior to starting the experiment, as documented in other studies.^12^ Conversely, U mobilized by DPA exhibited a distinct redox speciation with approximately half U(IV) and half U(VI) (53–55% U(IV), 45–47% U(VI)). Molinas et al. reported a similar result in work with ligand DPAEA (bis(pyridyl-6-methyl-2-carboxylate)-ethylamine), a structural analogue of DPA, which also contains pyridine and carboxylate functional groups. They confirmed the approximately 1:1 ratio in which U(IV) and U(VI) were found to relate to complexation of pentavalent U (U(V)) and subsequent disproportionation into U(IV) and U(VI) after acidification.^49^ Therefore, based on the structural similarity of the ligands and near-equivalent U(IV) and U(VI) proportions in acidified filtrates, it is likely that part of the U mobilized by DPA was in fact U(V) (resulting in approximately equal U(IV) and U(VI) concentrations); however, our method was not designed to distinguish U(V) from U(IV) and U(VI).

#### Competing Metals

**Figure 1:**
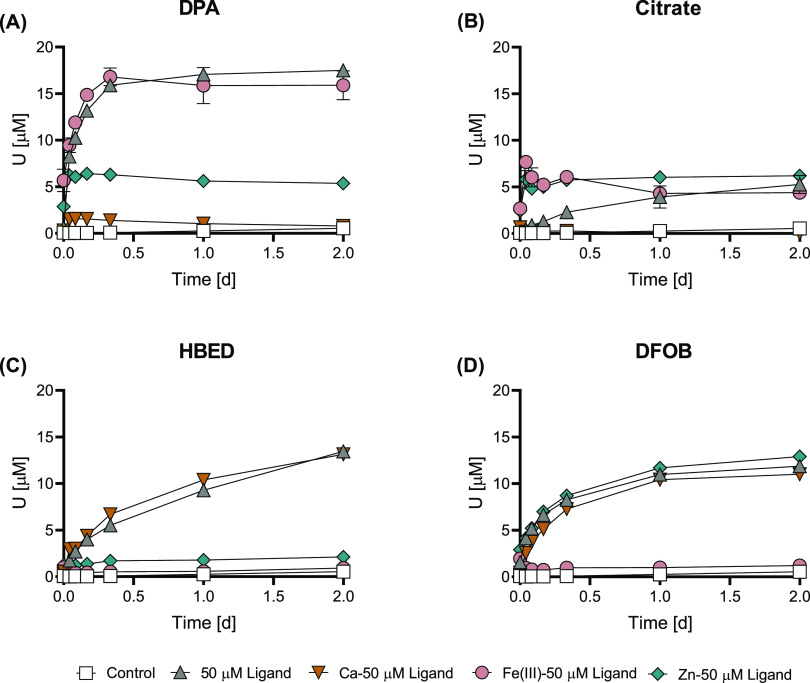
Effect of competing metals on ligand-induced U mobilization from noncrystalline U(IV) (300 μM U) by 50 μM (A) DPA, (B) citrate, (C) HBED, and (D) DFOB at pH 7.0. Calcium (2.2 mM) was added as CaCl_2_ as the electrolyte instead of NaCl for Ca treatments. Fe(III) and Zn were complexed by the ligands at equimolar concentrations (50 μM); Fe(III) was allowed to equilibrate for 12 h before filtering out uncomplexed precipitated Fe. Control treatments contained the same composition as other treatments with the exception of the ligand and competing metal. Data points show the mean (*n* = 2), with the ends of the error bars representing the measured values. Time *t* = 0 h corresponds to the moment of addition of an aliquot of noncrystalline U(IV) stock suspension to the solution containing pH buffer, electrolyte, ligand, and competing metal when applicable.

The effect of competing metals on ligand-induced U mobilization from noncrystalline U(IV) was assessed for three metals commonly found in the subsurface: Ca, Fe(III), and Zn. Our results illustrate that, depending on the metal–ligand combination, the effect could range from no significant change in U mobilization to enhanced U mobilization kinetics and >99% U mobilization inhibition compared to ligand-only treatments (Figures 1, S2, and S3).

Ca did not substantially affect the final mobilized U concentration in the presence of hexadentate ligands (HBED and DFOB) compared to ligand-only treatments (2 and 7% decrease in total U mobilized from Ca complexation, respectively) (Figure 1). This is consistent with PHREEQC calculations showing negligible complexation of Ca by HBED or DFOB under experimental conditions (Text S2 and Table S5). As complexation of Ca resulted in only minor deviations in U mobilization from ligand-only treatments, despite the free Ca concentration largely exceeding the free U concentration, it is expected that HBED and DFOB have a much higher affinity for U than for Ca. Conversely, for both tridentate ligands (DPA and citrate), complexation to Ca inhibited U mobilization by >95%. The diminished U mobilization for DPA and citrate due to Ca complexation can be attributed to a limited specificity for U in relation to structural features of the tridentate ligands.^29^ Aqueous speciation calculations carried out using PHREEQC (detailed in Text S2) by inputting citrate, Ca, and U(IV) suggest that citrate predominantly forms complexes with Ca (>95% of available citrate) in the experimental conditions with U present. This is consistent with the inhibition of U mobilization observed experimentally through competitive binding. For DPA, no U(IV) complexation constants were available for comparison.^50^ We note that U(IV) was input simply as aqueous U(IV) due to the lack of a binding constant for noncrystalline U(IV) in the literature. We note that in carbonate-rich systems, Ca-induced diminishment in U mobilization would be expected to occur to a lesser extent (especially where U(VI) is still present) due to the formation of soluble calcium uranyl carbonate complexes.

For the ligands where U mobilization was inhibited by Fe(III) complexation, Ca complexation was not inhibited and vice versa. For both HBED and DFOB, which form strong 1:1 complexes with Fe(III),^51,52^ the final mobilized U concentration decreased by >90% compared to the respective ligand-only treatments. This resulted in submicromolar levels of U for both ligands (with the exception of the initial sample, which exceeded 1 μM before dropping again) (Figure 1). The decrease in mobilized U can be attributed to either Fe complexation being thermodynamically favorable (Fe(III) outcompeting U(IV) for ligand complexation) or slow displacement kinetics compared to our experimental time scales.^53^ The affinity of DPA for Fe(III) is insufficient to maintain Fe dissolved.^54^ Therefore, only marginal Fe complexation occurred and the majority of 50 μM Fe precipitated out and was removed by filtration. The remaining dissolved Fe concentration was <5 μM prior to noncrystalline U(IV) addition (Figure S4). As a result, the difference in U mobilization between the DPA-only and Fe-DPA treatment was negligible: <8% lower dissolved U concentrations at 2 d in the Fe-DPA treatment. Similarly, Fe-citrate showed only a minor deviation from the ligand-only treatment regarding the final mobilized U concentration. However, U was mobilized substantially faster by Fe-citrate (Figure 1B).

While it is possible for Fe(III) to be reduced to Fe(II) while oxidizing U(IV),^55^ this is not expected to have played a large role in our experimental findings for DFOB, HBED, or DPA. This is because (I) DFOB and HBED form very stable complexes with Fe(III), which are not expected to be displaced, and (II) in the case of DPA, the majority of Fe was removed by filtration prior to the start of the experiment. For citrate, where an enhanced kinetic effect was observed, Fe(III)-facilitated redox reactions with reduced U(IV) cannot be ruled out (dissolved Fe(II) concentrations were not measured). Ion exchange chromatography was not possible in these treatments due to matrix interference effects.

Complexation of Zn exhibited specific effects on the ligand-induced mobilization of U from noncrystalline U(IV). Minor deviations in final mobilized U compared to ligand-only treatments were found for DFOB and citrate, while the final mobilized U concentration from DPA and HBED were partially inhibited by complexation of Zn (68 and 84% less final mobilized U compared to ligand-only treatments, respectively) (Figure 1). These findings align relatively well with the extent to which each ligand binds Zn in the absence of U.^51,56−58^ Specifically, the free Zn concentration in solution at pH 7.0 was predicted with PHREEQC for each ligand (1:1 metal-to-ligand ratio) in the current study. Model predictions showed that free Zn concentrations were the lowest for HBED (<2% of final Zn concentration), which had the highest inhibition of U mobilization. The highest free Zn concentration in solution was predicted for DFOB (>80%), for which no inhibitory effect was observed (free Zn concentrations for DPA and citrate were ∼30%). The oxidation state of the mobilized U at the end of the experiment (2 d) was not substantially affected by ligands initially complexing Zn (difference between U oxidation state from Zn–ligand and ligand-only treatments <8%) (Figure S5).

For the complexed Fe and Zn treatments with citrate, the kinetics of U mobilization was enhanced relative to the citrate-only treatment during the first 1 d. Subsequently, the U concentration stabilized and remained relatively constant at approximately the same final dissolved U concentration as the citrate-only treatment. Dissolved Fe and Zn concentrations rapidly decreased from the initial 50 to <25 μM within 1 h. This is consistent with a displacement reaction, whereby U mobilization is coupled to Fe and Zn immobilization e.g., through interactions between the metal–ligand complex and the biomass (Figures S4 and S6). Collins et al. demonstrated that Cd^2+^ adsorption to positively charged goethite (pH < 6.7) was more favorable in the presence of phosphate or sulfate by electrostatic interaction (by decreasing the positive charge of the surface).^59^ Applying a similar rationale, the kinetic effect on U mobilization from the negatively charged biomass in Fe- and Zn-citrate treatments might (partially) result from the decreased electrostatic repulsion compared to the free ligand (citrate). Fe-citrate and Zn-citrate^–^ are the dominant species and bear charges of 0 and −1, respectively, compared to the free ligand (citrate^3–^, −3 charge) at pH 7.0.

#### Humic Substances

**Figure 2:**
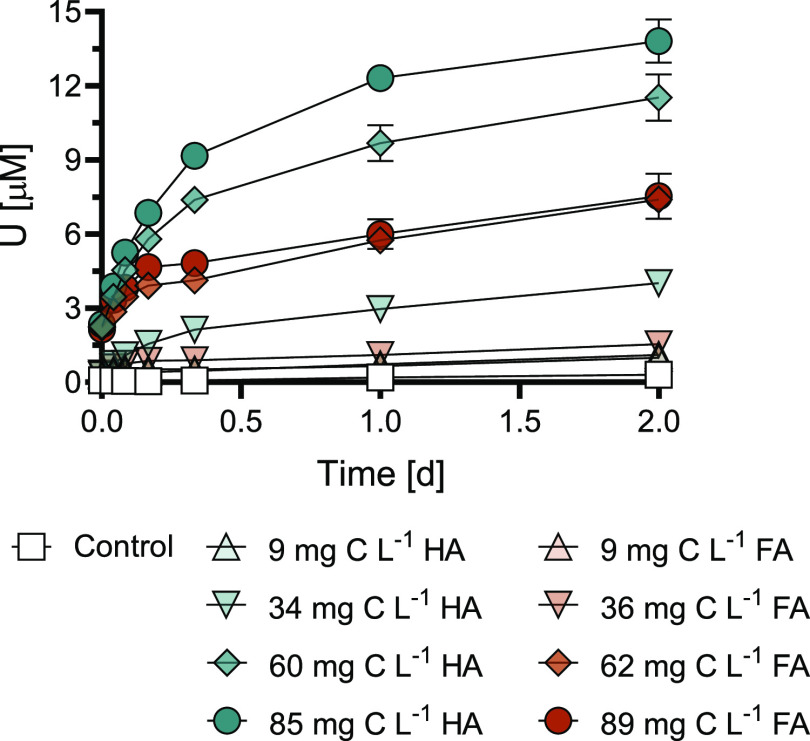
Humic substance-induced U mobilization from noncrystalline U(IV) (300 μM U) by HA or FA at pH 7.0. HA and FA were applied at concentrations of 9, 34, 60, and 85 mg C L^–1^ HA and 9, 36, 62, and 89 mg C L^–1^ FA. Control treatments contained the same composition as other treatments, with the exception of the HS. Data points show the mean (*n* = 2), with the ends of the error bars representing the measured values. Time *t* = 0 h corresponds to the moment of addition of an aliquot of noncrystalline U(IV) stock suspension to the solution containing pH buffer, electrolyte, and HA or FA.

Humic substances enhanced U mobilization from noncrystalline U(IV) at pH 7.0 (Figures 2 and S7–S8). Both 9 mg C L^–1^ HA and FA mobilized at least three times more U than the control at all measured time points, yet concentrations still remained below 1.1 μM. HA mobilized U to a greater extent and faster than FA in the concentration range from ∼30 to 90 mg C L^–1^ (total U mobilized as high as 4.6% in the 85 mg C L^–1^ HA treatment). This aligns with the higher stability constants of U(IV) complexes with HA than with FA^24,25^ and with a large binding site density of HA for both U(IV) and U(VI) compared to FA (more than a factor of 2).^25^ Furthermore, Luo et al. found that HA facilitated U mobilization from U-containing bioreduced field sediments to a greater extent than FA.^23^ HA has a higher phenolic content than FA (the estimate of the phenolic OH^–^ content is 45% higher for HA than for FA for Suwannee River Standard I; determined by titration).^40^ The higher phenolic content of HA would be expected to result in HA having a larger capacity for binding the hard acid U(IV) than FA.^60^ This could partially explain the greater U mobilization promoted by the reaction with HA than with FA. In total, 34 mg L^–1^ HA and 36 mg C L^–1^ FA mobilized similar proportions of U(IV) and U(VI) (Figure S8): 59% U(IV) and 41% U(VI) for HA and 64% U(IV) and 36% U(VI) for FA. Normalizing the 50 μM ligand concentration to moles of C yields concentrations ranging from 4 to 26 mg C L^–1^. Based on this and the results of Figures S1 and 2, HS are less effective at mobilizing U from noncrystalline U(IV) on a per mole of C basis. However, due to the large abundance of HS in water, soil, and sediments, they can still be an important contributor to U mobilization.^61^

#### Effects of Ca on U Mobilization by Humic Substances

**Figure 3:**
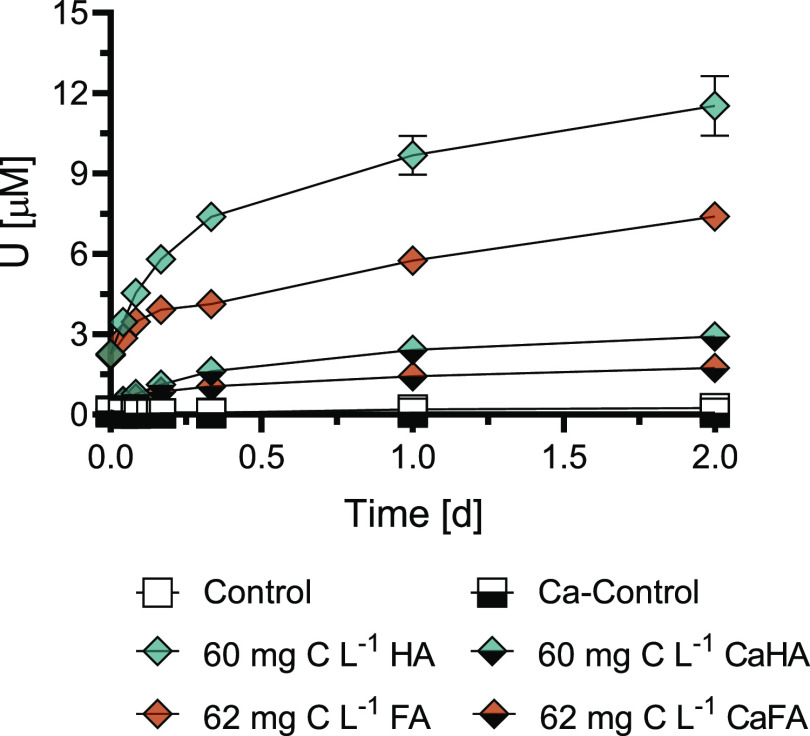
Effect of calcium complexation on humic substance-induced U mobilization from noncrystalline U(IV) (300 μM U) by 60 mg C L^–1^ HA or 62 mg C L^–1^ FA at pH 7.0 (10 mM MOPS). Control treatments contained the same composition as other treatments with the exception of the HS. Control was carried out with NaCl as the electrolyte, while Ca-control was carried out with CaCl_2_. Data points show the mean (*n* = 2), with the ends of the error bars representing the measured values. Time *t* = 0 h corresponds to the moment of addition of an aliquot of the noncrystalline U(IV) stock suspension to the solution containing pH buffer, electrolyte, and HA or FA.

Application of CaCl_2_ instead of NaCl as the electrolyte induced substantial inhibitory effects on U mobilization from noncrystalline U(IV) at ∼60 mg C L^–1^ HS. The addition of Ca resulted in 75% and 77% decreases in dissolved U concentrations with HA and FA after 2 d, respectively (Figure 3). Decreased U mobilization could be attributed to two factors that are investigated here: (1) competition between Ca and U for complexation by the functional groups of the HS and (2) Ca enhancing aggregation of HS, resulting in lower dissolved HA and FA concentrations.

Solutions of ∼60 mg C L^–1^ HS prepared in the absence of noncrystalline U(IV) showed that application of CaCl_2_ as the electrolyte compared to NaCl decreased DOC concentrations by only 19 and 6% for HA and FA, respectively. This decrease in the dissolved HS concentrations available for U mobilization resulted from Ca-induced HS coagulation (Figure S9). When noncrystalline U(IV) was added to the same solutions of ∼60 mg C L^–1^ HS with either NaCl or CaCl_2_ (in the absence of MOPS), similar removal of DOC from the solution was observed. In CaCl_2_-containing suspensions, 32% and 7% of the total DOC was removed from the solution compared to the NaCl treatment after 2 d for HA and FA, respectively (Figure S10). Losses in HS from the solution are attributed to aggregation and possibly sorption to biomass. The fraction of HA and FA lost from the solution due to Ca addition is considerably smaller than the decrease in mobilized U concentration (Figure 3). This implies that although aggregation plays a role (especially with HA), competitive effects from Ca have a larger effect on the smaller U mobilization.

#### Humic Substances and Anthropogenic or Biogenic Ligands Combined

**Figure 4:**
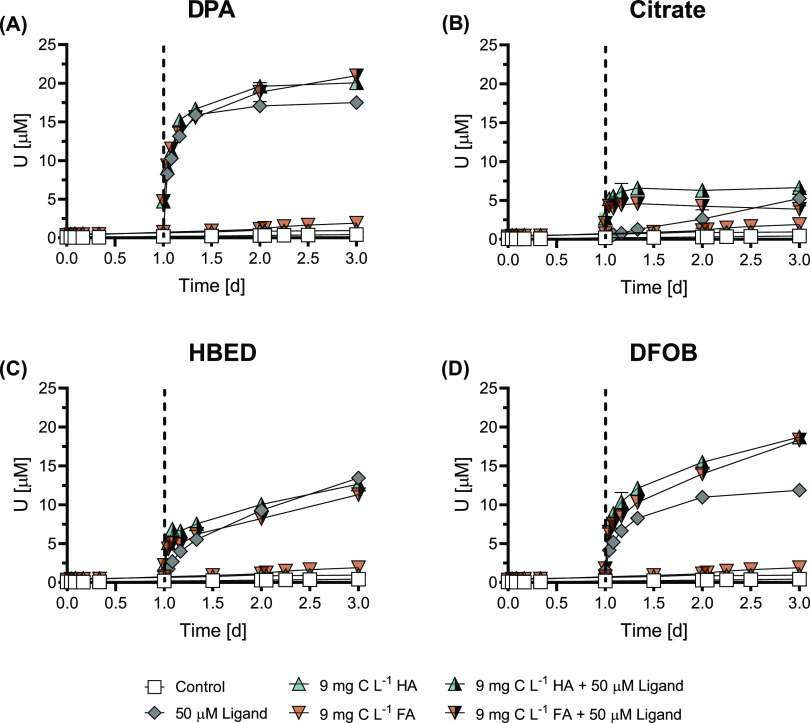
Effect of pre-equilibration of noncrystalline U(IV) (300 μM U) with 9 mg C L^–1^ HA or FA on ligand-induced U mobilization by 50 μM (A) DPA, (B) citrate, (C) HBED, and (D) DFOB at pH 7.0. Control treatments contained the same composition as other treatments, with the exception of the ligand and HS. Data points show the mean (*n* = 2), with the ends of the error bars representing the measured values. Time *t* = 0 h corresponds to the moment of addition of an aliquot of noncrystalline U(IV) stock suspension to the solution containing pH buffer, electrolyte, and HA or FA. The dashed line at *t* = 1.0 d corresponds to the moment of ligand addition.

The combined effects of HS and anthropogenic or biogenic ligands on ligand-induced U mobilization from noncrystalline U(IV) were also evaluated with the same ligands as above (Figures 4 and S11). During pre-equilibration of 9 mg C L^–1^ HA or FA with noncrystalline U(IV) for 1 d, dissolved U concentrations remained <0.75 μM until 50 μM ligand was added. Spiking of each respective ligand to reactors containing noncrystalline U(IV) pre-equilibrated with HS led to immediate U mobilization above concentrations in the ligand-only treatments. For all ligands, the combined effects of HS and anthropogenic or biogenic ligands resulted in a synergistic effect on U mobilization, but the time intervals during which synergistic mobilization occurred were ligand-specific. Synergistic U mobilization implies greater levels of U mobilization in the combined treatment compared to the summation of U mobilized in ligand-only and HS-only treatments. Specifically, in the case of citrate and HBED, synergistic levels of U mobilization occurred over the first day after ligand addition, while for DPA, synergistic levels occurred only after 1 d of ligand addition. DFOB, on the other hand, showed a lasting synergistic effect on U mobilization throughout the experiment. Experimental findings indicate that equilibrium was not reached by the end of the experiment in select cases (e.g., citrate-only). Longer experiments were not possible due to the risk of degrading biomass.^15^ The synergistic early mobilization rate for citrate and HBED could be attributed to HA and FA binding to the noncrystalline U(IV) biomass during the pre-equilibration step. Such binding, presumably through carboxyl functional groups of the HS,^40^ possibly labilized U(IV) and enhanced ligand complexation kinetics compared to U(IV) binding to phosphoryl groups within the biomass.^10^

For the DFOB and HS treatments, a synergistic effect on U mobilization was observed at all time points, with 6.2% of the total U being mobilized from either HA or FA by the end of the experiment. Previous studies have shown that siderophores and HS can form covalent linkages in addition to possible ternary complexes between covalently bound siderophores and HS with cadmium and plutonium.^62,63^ These processes could possibly contribute to the observed synergistic effect between HS and DFOB on U(IV) binding.

**Figure 5:**
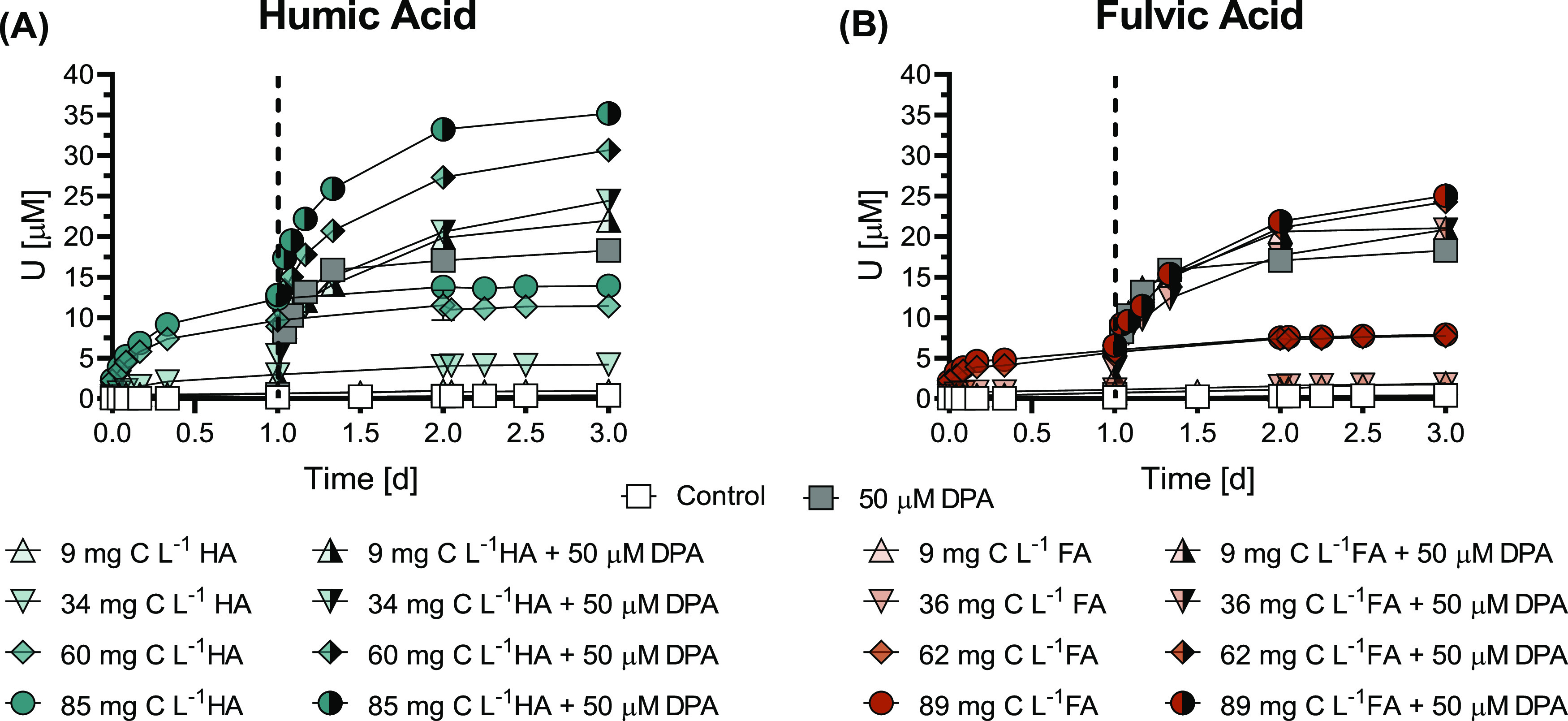
Effect of pre-equilibrated humic substance concentration on DPA-induced U mobilization from noncrystalline U(IV) (300 μM U) at pH 7.0. (A) HA or (B) FA was pre-equilibrated at concentrations of 9, 34, 60, and 85 mg C L^–1^ HA and 9, 36, 62, and 89 mg C L^–1^ FA prior to the addition of DPA to a final suspension concentration of 50 μM. Control treatments contained the same composition as other treatments with the exception of the ligand and HS. Data points show the mean (*n* = 2), with the ends of the error bars representing the measured values. Time *t* = 0 h corresponds to the moment of addition of an aliquot of noncrystalline U(IV) stock suspension to the solution containing pH buffer, electrolyte, and HA or FA. The dashed line at *t* = 1.0 d corresponds to the moment of DPA addition.

The effect of the pre-equilibrated HS concentration on U mobilization by a biogenic ligand was assessed with DPA for both Na and Ca as electrolyte cations. Results of experiments with Na as the electrolyte are provided in Figures 5 and S12, while Ca results are provided in Figure S13 and summarized in Text S3. Pre-equilibration of HA or FA with Na as the electrolyte cation exhibited a mostly additive effect on the dissolved U concentrations over the entire examined HS concentration range: ∼10–90 mg C L^–1^ (matching the concentrations in Figure 2). At the highest HS concentrations combined with DPA, dissolved U concentrations reached 35 and 25 μM for HA and FA, respectively.

The consistent additive effect on U mobilization from noncrystalline U(IV) by 50 μM DPA with increasing HS concentrations (compared to DPA-only and HS-only treatments) illustrates that the U pool available for complexation was not depleted up to ∼90 mg C L^–1^ HS (>12% of the total U mobilized). Hence, in environments where DOC concentrations are high, e.g., peat lands, the contribution of HS to U mobilization is likely to be in the same order or larger than the contribution of anthropogenic and biogenic ligands.

## Environmental Implications

Anthropogenic and biogenic ligands can mobilize U from U(IV) phases.^12,15,16^ Yet, in the environment, organic ligands encounter competition toward binding U (or other target metals) from the constituents of complex soil and sediment matrices. This study demonstrates that competitive complexation of other metals and U complexation by HS can affect the relative rate and total extent to which U is mobilized from noncrystalline U(IV). These findings contribute to the mechanistic understanding of the impact of organic ligands in the subsurface toward mobilizing U from U(IV) phases.

Competing metals (Ca, Fe(III), and Zn) had greatly varied effects on U mobilization by anthropogenic and biogenic ligands, ranging from near-complete inhibition to no inhibitory effect and enhanced kinetic effects, depending on the metal–ligand combination. These results illustrate how ligand specificity can govern the mobilization of a metal of interest in sediments rich in other metals competing for complexation. Conversely, humic substances (HA, FA), constituting a large component of natural organic matter (NOM), mobilized appreciable levels of U from noncrystalline U(IV) at pH 7.0. While Ca inhibited U mobilization by HS through coagulation and competitive binding, U mobilization by anthropogenic and biogenic ligands was enhanced by the presence of HS. These results underscore the increased risk of U mobilization, where U deposition occurs (e.g., via bioreduction) in organic-rich environments (e.g., mining polluted wetlands).^26,64^

For accurate predictive measures of the risk of ligand-induced U mobilization, competitive complexation of other metals and mobilization and labilization of reduced U(IV) by HS should be considered. Notably, Ca and Fe(III) were shown to have the greatest impact on hindering U mobilization, depending on the ligand. Conversely, FA and, to a greater extent, HA were shown to increase U mobilization. As such, results from this study provide valuable insights into the planning of U-contaminated site characterization and ensuing remediation efforts. Future challenges include exploring the effect of additional environmental parameters (e.g., pH) and quantitatively analyzing the effects established in this study in soils and subsurface sediments, e.g., in flow-through experiments, to draw closer to replicating field conditions.

## Supporting Information Available

Description of the ion exchange chromatography method, approach for aqueous speciation calculations, and experiments containing Ca, HS, and DPA; Widdel low-phosphate medium composition; overview of experimental treatments; thermodynamic stability constants; overview of aqueous speciation calculation results from PHREEQC modeling; ligand-induced U mobilization from noncrystalline U(IV); summary of total U mobilized in treatments from Figure 1; zoom in view of Figure 1; dissolved Fe and Zn concentrations from corresponding competing metal treatments; ion exchange chromatography results from Zn competing metal treatment; summary of total U mobilized in treatments from Figure 2; ion exchange chromatography results from 34/36 mg C L^–1^ HA/FA treatments; DOC results from 60/62 mg C L^–1^ solutions and experiments with noncrystalline U(IV) in the absence of MOPS; summary of total U mobilized in treatments from Figure 4 and 5; and overview of calcium effects on U mobilization from noncrystalline U(IV) combined with HS and DPA (PDF)
