# Supplementary material for: Effect of Competing Metals and Humic Substances on Uranium Mobilization from Noncrystalline U(IV) Induced by Anthropogenic and Biogenic Ligands
Source: Environ Sci Technol. 2023 Oct 11;57(42):16006–15. doi: 10.1021/acs.est.3c01705 (PMC10603774; doi:10.1021/acs.est.3c01705)
Supplement: Supplementary file 1 — es3c01705_si_001.pdf [file es3c01705_si_001.pdf]

Effect of Competing Metals and Humic Substances on Uranium Mobilization from Noncrystalline U(IV) Induced by Anthropogenic and Biogenic Ligands

Kyle J. Chardi[a], Walter D. C. Schenkeveld[b*], Naresh Kumar[b*], Daniel E. Giammar[c], Stephan M. Kraemer[a]

[a] Centre for Microbiology and Environmental Systems Science, Department for Environmental Geosciences, University of Vienna, Josef-Holaubek-Platz 2 1090 Vienna, Austria
[b] Soil Chemistry and Chemical Soil Quality Group, Wageningen University and Research, Droevendaalsesteeg 3, 6708 PB Wageningen, the Netherlands
[c] Department of Energy, Environmental, and Chemical Engineering, One Brookings Drive, Washington University, St. Louis, Missouri 63130, United States

* Corresponding author: walter.schenkeveld@wur.nl

* Corresponding author: naresh.kumar@wur.nl

Supporting Information

23 pages

3 text sections

5 tables

13 figures

This supporting information contains the following:

Text S1. Ion exchange chromatography

Text S2. Approach to aqueous speciation calculations

Text S3. Combined effects of Ca, humic substances, and DPA on ligand-induced U mobilization

Table S1. Basal medium composition of Widdel Low Phosphate (WLP) used for the synthesis of noncrystalline U(IV).

Table S2. Overview of conditions for competing metal experiments (Figure 1).

Table S3 Overview of all experimental treatments and associated figures.

Table S4. Thermodynamic stability constants (T = 25° C, I = 0) for aqueous and solid phase reactions as used in PHREEQC.

Table S5. Overview of aqueous speciation predictions carried out in PHREEQC for competing metal complexation with anthropogenic and biogenic ligands. Individual models were run for each respective ligand at 50 µM and competing metal (no U due to lack of complexation constants with various ligands). For both Fe(III) and Zn models 50 µM of metal were added while for Ca 2.2 mM was added in place of NaCl as the ionic strength contributor. The modelling approach is summarized in Text S2 and the relevant stability constants are summarized in Table S3.

Figure S1. Ligand-induced U mobilization from noncrystalline U(IV) (300 µM U) by 0.5, 5, and 50 µM (A) DPA, (B) citrate, (C) HBED, and (D) DFOB at pH 7.0. Ligand-free control treatments had the same composition as other treatments except for the ligand addition. Data points show the mean (n = 2) with the ends of the error bars representing the measured values. Time t = 0 h corresponds to the moment of addition of an aliquot of noncrystalline U(IV) stock suspension to the solution containing pH buffer, electrolyte, and ligand. Bar plots represent the solution concentrations of the U redox species determined by ion exchange chromatography at the 2 d timepoint for each corresponding treatment.

Figure S2. Summary of total U mobilized from noncrystalline U(IV) (300 μM U) in competitive displacement experiments with Ca, Fe(III), and Zn complexed to 50 μM DPA, citrate, HBED, and DFOB at pH 7.0 corresponding to Figure 1.

Figure S3. Effect of competing metals on ligand-induced U mobilization from noncrystalline U(IV) (300 μM U) by 50 μM (A) DPA, (B) citrate, (C) HBED, and (D) DFOB, at pH 7.0 (Figure 1) plotted on a smaller vertical axis scale. 2.2 mM calcium was added as $CaCl_2$ as electrolyte instead of NaCl for Ca treatments. Fe(III) and Zn were complexed by the ligands at equimolar concentrations (50 μM); Fe(III) was allowed to equilibrate for 12 hours before filtering out uncomplexed precipitated Fe. Control treatments contained the same composition as other treatments with the exception of the ligand and competing metal. Data points show the mean (n = 2) with the ends of the error bars representing the measured values. Time t = 0 h corresponds to the moment of addition of an aliquot of noncrystalline U(IV) stock suspension to the solution containing pH buffer, electrolyte, ligand, and competing metal when applicable.

Figure S4. Dissolved U and Fe concentrations in iron competitive displacement experiments with 50 μM DPA, citrate, HBED, and DFOB from noncrystalline U(IV) (300 μM U), at pH 7.0. Data points show the mean (n = 2) with the ends of the error bars representing the measured values. Time t = 0 corresponds to the moment of addition of an aliquot of noncrystalline U(IV) stock suspension to the solution containing pH buffer, electrolyte, ligand, and competing metal when applicable; corresponding with Figure 1.

Figure S5. Effect of 50 μM Zn complexation on ligand-induced U mobilization from noncrystalline U(IV) (300 μM U) by 50 μM DPA, citrate, HBED, and DFOB at 7.0. Control treatments contained the same composition as other treatments with the exception of the ligand and competing metal. Data points show the mean (n = 2) with the ends of the error bars representing the measured values. Time t = 0 h corresponds to the moment of addition of an aliquot of noncrystalline U(IV) stock suspension to the solution containing pH buffer, electrolyte, ligand, and competing metal when applicable. Bar plots correspond with filtered aqueous U oxidation state determination by ion

exchange chromatography at the 2 d timepoint for each corresponding treatment and color; corresponding with Figure 1.

Figure S6. Dissolved U and Zn concentrations in zinc competitive displacement experiments with 50 μM DPA, citrate, HBED, and DFOB from noncrystalline U(IV) (300 μM U), at 7.0. Control treatments contained the same composition as other treatments with the exception of the ligand. Data points show the mean (n = 2) with the ends of the error bars representing the measured values. Time t = 0 corresponds to the moment of addition of an aliquot of noncrystalline U(IV) stock suspension to the solution containing pH buffer, electrolyte, ligand, and competing metal when applicable; corresponding with Figure 1.

Figure S7. Summary of total U mobilized by HA and FA from noncrystalline U(IV) (300 μM U) at concentrations of 9, 34, 60, and 85 mg C L^{-1} HA and 9, 36, 62, and 89 mg C L^{-1} FA at pH 7.0 corresponding with Figure 2.

Figure S8. Humic substance-induced U mobilization from noncrystalline U(IV) (300 μM U) by 34 mg C L^{-1} HA or 36 mg C L^{-1} FA at 7.0. Control treatments contained the same composition as other treatments with the exception of the HS. Data points show the mean (n = 2) with the ends of the error bars representing the measured values. Time t = 0 h corresponds to the moment of addition of an aliquot of noncrystalline U(IV) stock suspension to the solution containing pH buffer, electrolyte, and humic substance. Bar plots correspond with filtered aqueous U oxidation state determination by ion exchange chromatography at the 2 d timepoint for each corresponding treatment and color.

Figure S9. DOC measurement from 60 mg C L^{-1} HA and 62 mg C L^{-1} FA filtered (0.45 μm, nylon) solutions in either NaCl or $CaCl_2$ solutions at concentrations equal to 10 mM ionic strength.

Figure S10. DOC concentrations over time from 60 mg C L^{-1} HA or 62 mg C L^{-1} FA prepared in either NaCl or $CaCl_2$ before addition to noncrystalline U(IV) (300 μM U) at pH 7.0 (± 0.3 pH units in absence of organic buffer). Electrolyte solutions of NaCl and $CaCl_2$ were prepared to account for a final ionic strength of 10 mM. Control treatments contained the same composition as other treatments with the exception of the HS.

Time t = 0 h corresponds to the moment of addition of an aliquot of noncrystalline U(IV) stock suspension to the solution containing electrolyte, and humic substance.

Figure S11. Summary of total U mobilized from pre-equilibration of 9 mg C L^{-1} HA or FA with noncrystalline U(IV) (300 μM U) followed by addition of 50 μM DPA, DFOB, HBED, and citrate, at pH 7.0 corresponding to Figure 4.

Figure S12. Summary of total U mobilized from noncrystalline U(IV) (300 μM U) by 9, 34, 60, and 85 mg C L^{-1} HA and 9, 36, 62, and 89 mg C L^{-1} FA individually and in combination with 50 μM DPA addition at pH 7.0.

Figure S13. Overview of calcium effects on U mobilization from noncrystalline U(IV) (300 μM U) by control, DPA, humic substance, and combined treatments at pH 7.0. Control treatments contained the same composition as other treatments with the exception of the ligand and HS. Control was carried out with NaCl as electrolyte while Ca-control was carried out with $CaCl_2$. Data points show the mean (n = 2) with the ends of the error bars representing the measured values.

Text S1. Ion exchange chromatography

Ion exchange chromatography was used to determine the dissolved U(IV) and U(VI) concentrations at the end of experiments (2 d) for selected treatments in accordance with Stoliker et al. and Wang et al.[1, 2] Polypropylene chromatography columns (Poly-Prep, Bio-Rad) were packed under anoxic conditions with Dowex 1 x 8 (200 – 400 mesh) anion exchange resin with chloride adsorbed. Samples were first filtered (0.2 μm, cellulose acetate) before stabilization in 4.5 M HCl. Samples were then spiked into pre-treated (10 pore volumes 0.1 M HCl, 10 pore volumes of 4.5 M HCl) resin-packed columns and the U redox species were selectively eluted with differing HCl concentrations. U(VI) was eluted by 10 pore volumes of 0.1 M HCl and subsequently U(IV) was eluted by 10 pore volumes of 4.5 M HCl.[1, 2] All samples were further diluted in 1% HNO_3 before analysis by ICP-MS.

Text S2. Approach to aqueous speciation calculations

Aqueous speciation predictions were carried out using PHREEQC to determine the affinity of each respective ligand towards each competing metal. Ligand, metal, and electrolyte compositions and concentrations as summarized in Figure 1 and Table S2 were used in models. Due to the lack of complexation constants for several of the ligands used in the study, U was not added in the results tabulated in Table S2. For citrate, where U(IV) complexation constants are available, additional models were run by inputting the total U suspension concentration into solution as 300 μM U(IV). Due to the absence of a binding constant of U for noncrystalline U(IV), it was not possible to add an equilibrium phase into models. Possible solid U phases were not allowed to precipitate.

Text S3. Combined effects of Ca, humic substances, and DPA on ligand-induced U mobilization

The combined effects of Ca (2.2 mM) and humic substance (60 mg C L^{-1} HA, 62 mg C L^{-1} FA) pre-equilibration on ligand-induced U mobilization was probed with 50 μM DPA at pH 7.0 (Figure S12). As Ca was already shown to significantly inhibit the ability of DPA as well as HA and FA to mobilize U, the combined effects still resulted in significant inhibition despite having both DPA and HA or FA present. Notably, DPA in the presence of HA or FA in addition to Ca results in either similar or lower mobilized U concentrations than the DPA-only treatment. This highlights the large role competing metals can play in diminishing specific metal mobilization by anthropogenic and biogenic ligands in addition to humic substances.

Table S1. Basal medium composition of Widdel Low Phosphate (WLP) used for the synthesis of noncrystalline U(IV).

Compound	WLP (mM)
$CaCl_2 \cdot 2H_2O$	0.68
KCl	6.71
KH_2PO_4	0.22
$MgCl_2 \cdot 6H_20$	2.46
NaCl	85.56
NH_4Cl	4.67
$NaHCO_3$	30
PIPES	20
pH	7.3

Table S2. Overview of conditions for competing metal experiments (Figure 1).

Competing Metal	Chemical	Final Concentration	Ionic Strength Electrolyte
Ca	$CaCl_2$	2.2 mM	2.2 mM $CaCl_2$
Fe(III)	$FeCl_3$	50 μM	6.6 mM NaCl
Zn	$ZnCl_2$	50 μM	6.6 mM NaCl

Table S3. Overview of all experimental treatments and associated figures.

Treatment	Ligand/HS	Concentrations	Competing metal	Figure
Ligand	DPA, Citrate, HBED, DFOB	0.5, 5, 50 μM	-	Figure S1
Competing metal	DPA, Citrate, HBED, DFOB	50 μM	2.2 mM Ca, 50 μM Fe(III), 50 μM Zn	Figure 1, Figure S2 - S6
HS	HA, FA	9, 34/36, 60/62, 85/89 mg C L^{-1} HA/FA	-	Figure 2, Figure S7
HS + Ca	HA, FA	60/62 mg C L^{-1} HA/FA	2.2 mM Ca	Figure 3, Figure S9, S10
HS + ligand	HA, FA + DPA, Citrate, HBED, DFOB	9 mg C L^{-1} HS + 50 μM ligand	-	Figure 4, Figure S11
HS concentration + ligand	HA, FA + DPA	9, 34/36, 60/62, 85/89 mg C L^{-1} HA/FA + 50 μM DPA	-	Figure 5, Figure S12
HS concentration + ligand + Ca	HA, FA + DPA	60/62 mg C L^{-1} HA/FA + 50 μM DPA	2.2 mM Ca	Figure S13

Table S4. Thermodynamic stability constants (T = 25° C, I = 0) for aqueous and solid phase reactions as used in PHREEQC.

Reaction	Log K	Source
**Citrate Hydrolysis**		
$Citrate^{3-} + H^+ \rightleftharpoons HCitrate^{2-}$	6.4	1
$Citrate^{3-} + 2H^+ \rightleftharpoons H_2Citrate^-$	11.2	1
$Citrate^{3-} + 3H^+ \rightleftharpoons H_3Citrate_{(aq)}$	14.3	1
**U(IV)-Citrate Complexation**		
$Citrate^{3-} + U^{4+} \rightleftharpoons UCitrate^+$	12.8	2
$2Citrate^{3-} + U^{4+} \rightleftharpoons U(Citrate)_2^{2+}$	19.5	1
**Ca-Citrate Complexation**		
$Citrate^{3-} + Ca^{2+} \rightleftharpoons CaCitrate^-$	4.73	3
$Citrate^{3-} + Ca^{2+} + H^+ \rightleftharpoons CaHCitrate_{(aq)}$	3.02	3
$Citrate^{3-} + Ca^{2+} + 2H^+ \rightleftharpoons CaH_2Citrate^+$	1.29	3
**Fe(III)-Citrate Complexation**		
$Citrate^{3-} + Fe^{3+} \rightleftharpoons FeCitrate_{(aq)}$	13.17	1
$HCitrate^{2-} + Fe^{3+} \rightleftharpoons FeHCitrate^+$	8.02	1
**Zn-Citrate Complexation**		
$Citrate^{3-} + Zn^{2+} \rightleftharpoons ZnCitrate^-$	6.21	3
$2Citrate^{3-} + Zn^{2+} \rightleftharpoons Zn(Citrate)_2^{4-}$	7.4	3
$Citrate^{3-} + Zn^{2+} + H^+ \rightleftharpoons ZnHCitrate_{(aq)}$	10.2	3
$Citrate^{3-} + Zn^{2+} + 2H^+ \rightleftharpoons ZnH_2Citrate^+$	12.84	3
**DPA Hydrolysis**		
$DPA^{2-} + H^+ \rightleftharpoons HDPA^-$	4.53	4
$DPA^{2-} + 2H^+ \rightleftharpoons H_2DPA_{(aq)}$	6.60	4
$DPA^{2-} + 3H^+ \rightleftharpoons H_3DPA^+$	8.62	4
**Ca-DPA Complexation**		
$DPA^{2-} + Ca^{2+} \rightleftharpoons CaDPA_{(aq)}$	4.39	5
$2DPA^{2-} + Ca^{2+} \rightleftharpoons Ca(DPA)_2^{2-}$	6.64	5
**Fe(III)-DPA Complexation**		
$DPA^{2-} + Fe^{3+} \rightleftharpoons FeDPA^+$	8.84	4
$2DPA^{2-} + Fe^{3+} \rightleftharpoons Fe(DPA)_2^{2-}$	14.82	4
**Zn-DPA Complexation**		
$DPA^{2-} + Zn^{2+} \rightleftharpoons ZnDPA_{(aq)}$	6.4	6
$2DPA^{2-} + Zn^+ \rightleftharpoons Zn(DPA)_2^{2-}$	12.1	6
**HBED Hydrolysis**		
$HBED^{4-} + H^+ \rightleftharpoons HHBED^{3-}$	12.6	7
$HBED^{4-} + 2H^+ \rightleftharpoons H_2HBED^{2-}$	23.6	7

$HBED^{4-} + 3H^+ \rightleftharpoons H_3HBED^-$	32.04	7
$HBED^{4-} + 4H^+ \rightleftharpoons H_4HBED_{(aq)}$	36.76	7
$HBED^{4-} + 5H^+ \rightleftharpoons H_5HBED^+$	39.29	7
$HBED^{4-} + 6H^+ \rightleftharpoons H_6HBED^{2+}$	40.99	7
**Ca-HBED Complexation**		
$HBED^{4-} + Ca^{2+} \rightleftharpoons CaHBED^{2-}$	9.29	8
$HBED^{4-} + Ca^{2+} + H^+ \rightleftharpoons CaHHBED^-$	8.69	8
$HBED^{4-} + Ca^{2+} + 2H^+ \rightleftharpoons CaH_2HBED_{(aq)}$	16.19	8
**Fe(III)-HBED Complexation**		
$HBED^{4-} + Fe^{3+} \rightleftharpoons FeHBED^-$	39.01	9
$HBED^{4-} + Fe^{3+} + H^+ \rightleftharpoons FeHHBED_{(aq)}$	40.52	9
**Zn-HBED Complexation**		
$HBED^{4-} + Zn^{2+} \rightleftharpoons ZnHBED^{2-}$	18.37	8
$HBED^{4-} + Zn^{2+} + H^+ \rightleftharpoons ZnHHBED^-$	8.17	8
$HBED^{4-} + Zn^{2+} + 2H^+ \rightleftharpoons ZnH_2HBED_{(aq)}$	14.0	8
**DFOB Hydrolysis**		
$DFOB^{3-} + H^+ \rightleftharpoons HDFOB^{2-}$	11.48	1
$DFOB^{3-} + 2H^+ \rightleftharpoons H_2DFOB^-$	21.46	1
$DFOB^{3-} + 3H^+ \rightleftharpoons H_3DFOB_{(aq)}$	30.66	1
$DFOB^{3-} + 4H^+ \rightleftharpoons H_4DFOB^+$	38.96	1
**Ca-DFOB Complexation**		
$HDFOB^{2-} + Ca^{2+} \rightleftharpoons CaHDFOB_{(aq)}$	3.52	1
**Fe(III)-DFOB Complexation**		
$HDFOB^{2-} + Fe^{3+} \rightleftharpoons FeHDFOB^+$	32.02	1
$HDFOB^{2-} + Fe^{3+} + H^+ \rightleftharpoons FeH_2DFOB^{2+}$	32.7	1
**Zn-DFOB Complexation**		
$DFOB^{3-} + Zn^{2+} \rightleftharpoons ZnDFOB^-$	12.03	10
$DFOB^{3-} + Zn^{2+} + H^+ \rightleftharpoons ZnHDFOB_{(aq)}$	22.10	10
$DFOB^{3-} + Zn^{2+} + 2H^+ \rightleftharpoons ZnH_2DFOB^+$	29.85	10

(1) Smith and Martell, 2004[3]
(2) Bonin et al. 2008[4]
(3) Gustafsson et al. 2011[5]
(4) Bombi et al. 2009[6]
(5) Tang et al. 1968[7]
(6) Suzuki et al. 1957[8]
(7) Motekaitis et al. 1990[9]
(8) L'Eplattenier et al. 1967[10]
(9) Ma et al. 1994[11]
(10) Northover et al. 2022[12]

Table S5. Overview of aqueous speciation predictions carried out in PHREEQC for competing metal complexation with anthropogenic and biogenic ligands. Individual models were run for each respective ligand at 50 µM and competing metal (no U due to lack of complexation constants with various ligands). For both Fe(III) and Zn models 50 µM of metal were added while for Ca 2.2 mM was added in place of NaCl as the ionic strength contributor. The modelling approach is summarized in Text S2 and the relevant stability constants are summarized in Table S3.

**Ligand**		**Ca**	**Fe(III)**	**Zn**
**DPA**	**Dominant species**	CaDPA	$Fe(DPA)_2^-$	ZnDPA
	**Concentration [M]**	4.8E-05	1.5E-08	2.2E-05
**Citrate**	**Dominant species**	$Ca(Citrate)^-$	Fe(Citrate)	$Zn(Citrate)^-$
	**Concentration [M]**	4.4E-05	1.9E-05	3.5E-05
**HBED**	**Dominant species**	$CaHBED^{-2}$	$Fe(HBED)^-$	$ZnHBED^{-2}$
	**Concentration [M]**	1.7E-09	5.0E-05	4.0E-05
**DFOB**	**Dominant species**	$CaDFOB^-$	$FeH(DFOB)^+$	$ZnH_2(DFOB)^+$
	**Concentration [M]**	3.0E-15	5.0E-05	8.0E-06

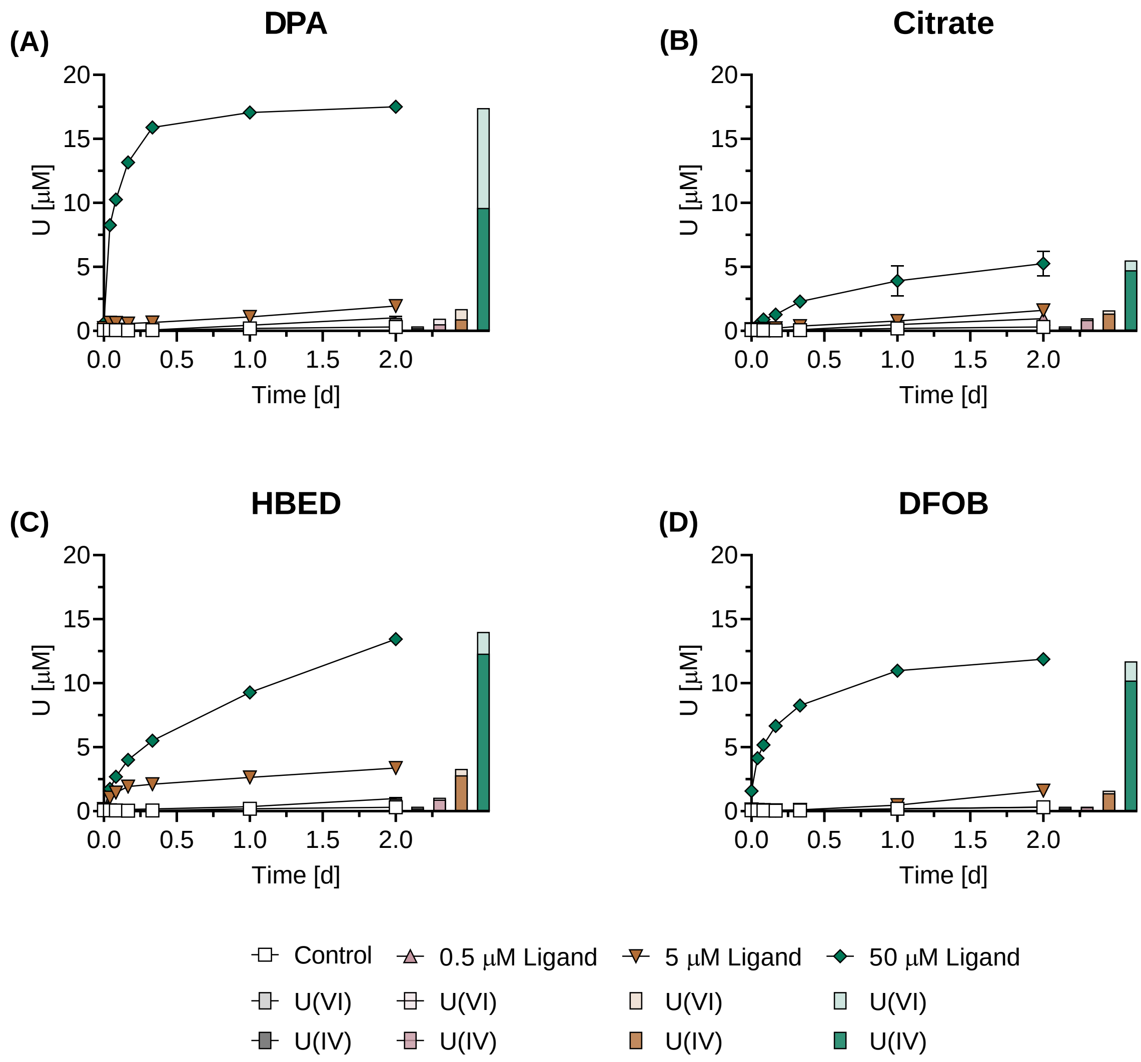

Figure S1. Ligand-induced U mobilization from noncrystalline U(IV) (300 µM U) by 0.5, 5, and 50 µM (A) DPA, (B) citrate, (C) HBED, and (D) DFOB at pH 7.0. Ligand-free control treatments had the same composition as other treatments except for the ligand addition. Data points show the mean (n = 2) with the ends of the error bars representing the measured values. Time t = 0 h corresponds to the moment of addition of an aliquot of noncrystalline U(IV) stock suspension to the solution containing pH buffer, electrolyte, and ligand. Bar plots correspond with filtered aqueous U oxidation state determination by ion exchange chromatography at the 2 d timepoint for each corresponding treatment and color.

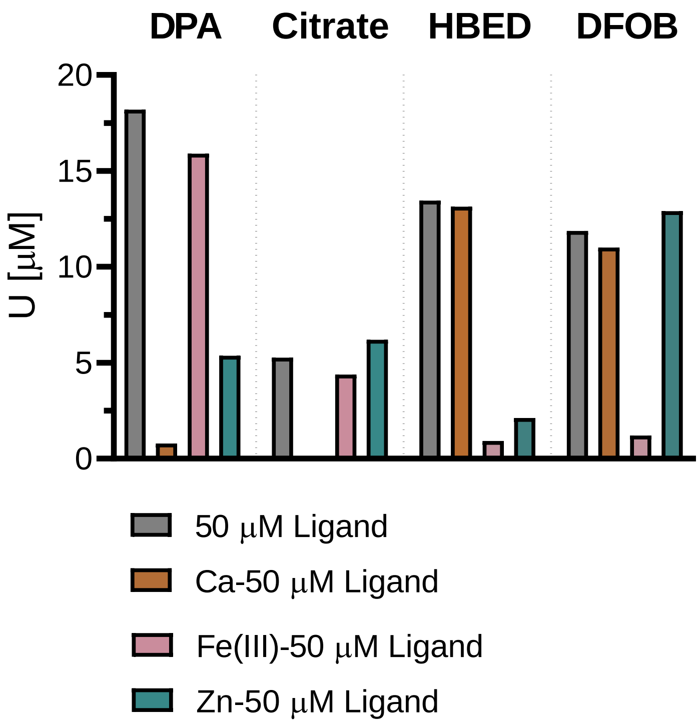

Figure S2. Summary of total U mobilized (2 d) from noncrystalline U(IV) (300 μM U) in competitive displacement experiments with Ca, Fe(III), and Zn complexed to 50 μM DPA, citrate, HBED, and DFOB at pH 7.0 corresponding to Figure 1.

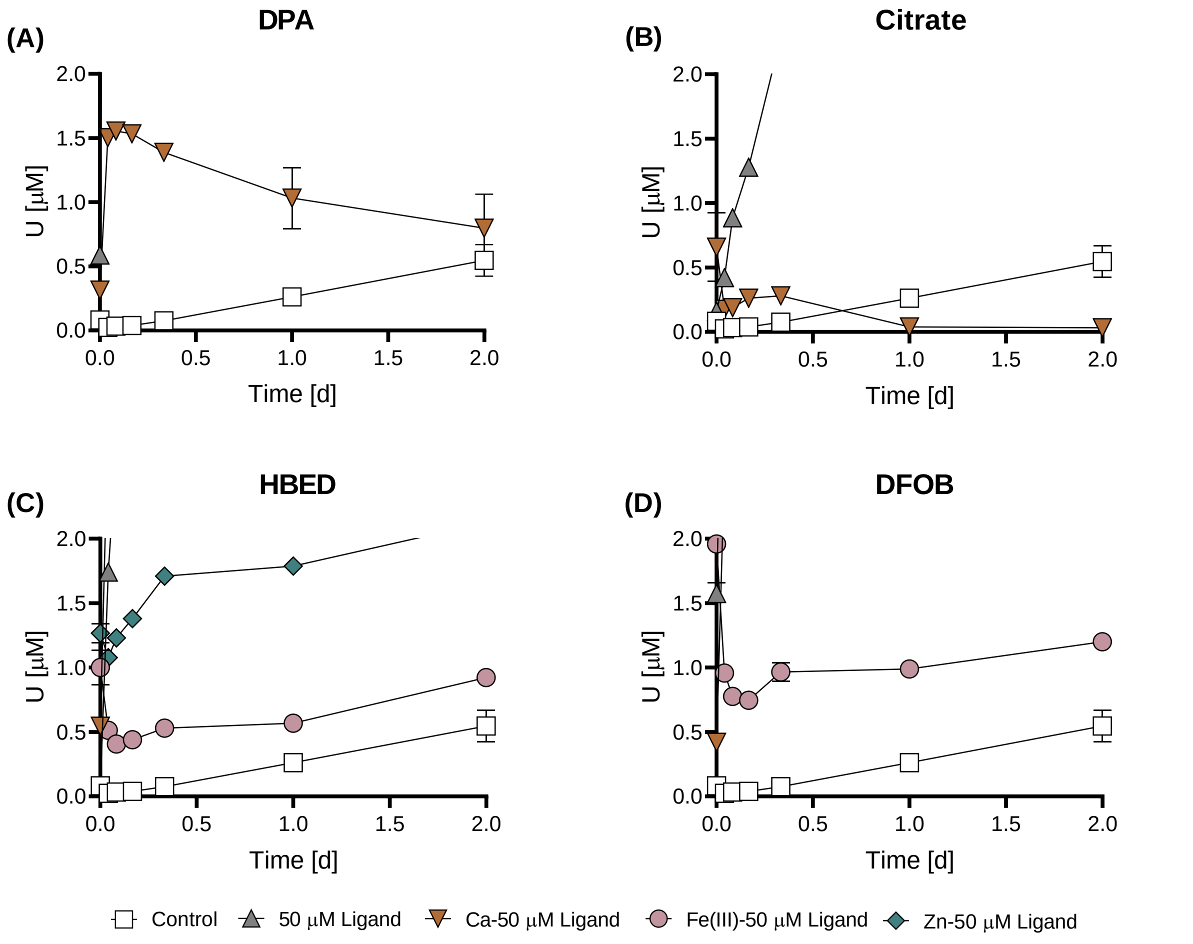

Figure S3. Effect of competing metals on ligand-induced U mobilization from noncrystalline U(IV) (300 µM U) by 50 µM (A) DPA, (B) citrate, (C) HBED, and (D) DFOB, at pH 7.0 (Figure 1) plotted on a smaller vertical axis scale. 2.2 mM calcium was added as $CaCl_2$ as electrolyte instead of NaCl for Ca treatments. Fe(III) and Zn were complexed by the ligands at equimolar concentrations (50 µM); Fe(III) was allowed to equilibrate for 12 hours before filtering out uncomplexed precipitated Fe. Control treatments contained the same composition as other treatments with the exception of the ligand and competing metal. Data points show the mean (n = 2) with the ends of the error bars representing the measured values. Time t = 0 h corresponds to the moment of addition of an aliquot of noncrystalline U(IV) stock suspension to the solution containing pH buffer, electrolyte, ligand, and competing metal when applicable.

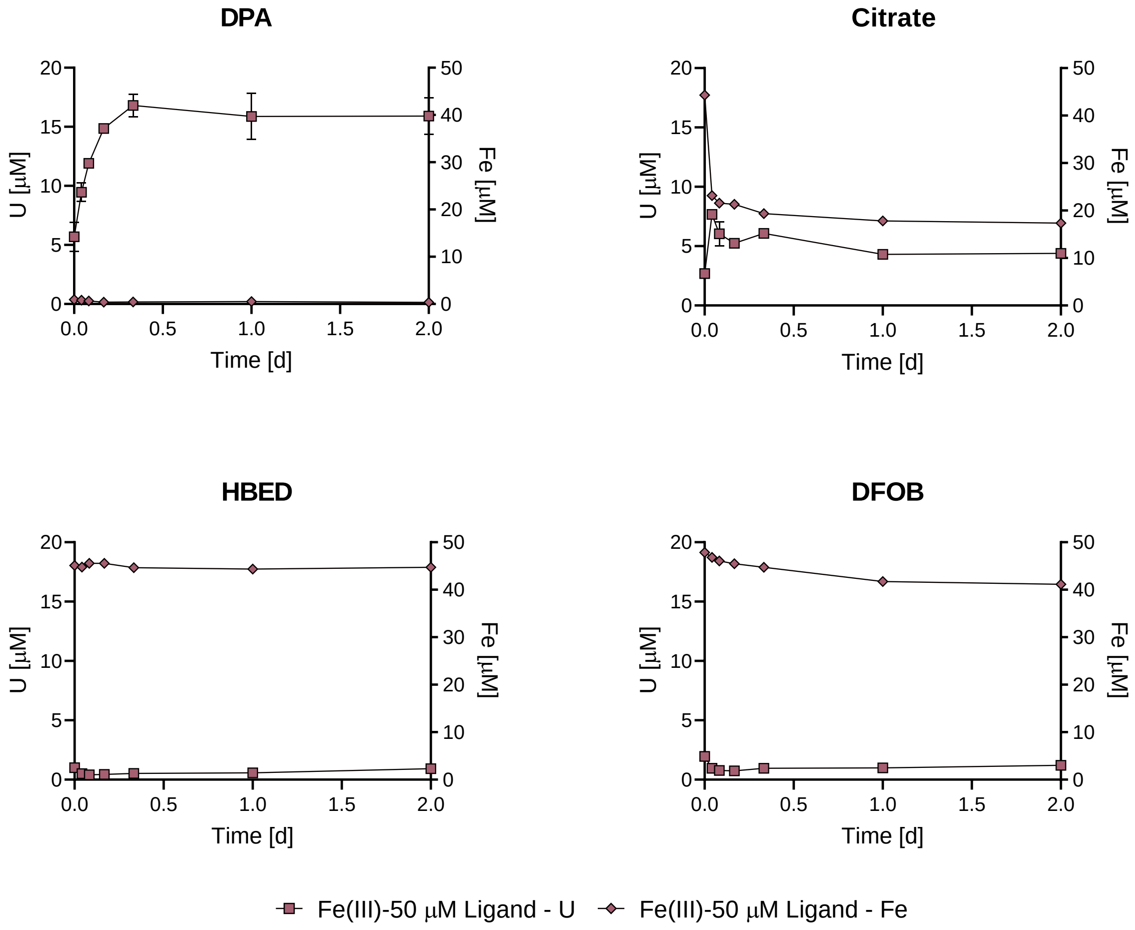

Figure S4. Dissolved U and Fe concentrations in iron competitive displacement experiments with 50 μM DPA, citrate, HBED, and DFOB from noncrystalline U(IV) (300 μM U), at pH 7.0. Data points show the mean (n = 2) with the ends of the error bars representing the measured values. Time t = 0 corresponds to the moment of addition of an aliquot of noncrystalline U(IV) stock suspension to the solution containing pH buffer, electrolyte, ligand, and competing metal when applicable; corresponding with Figure 1.

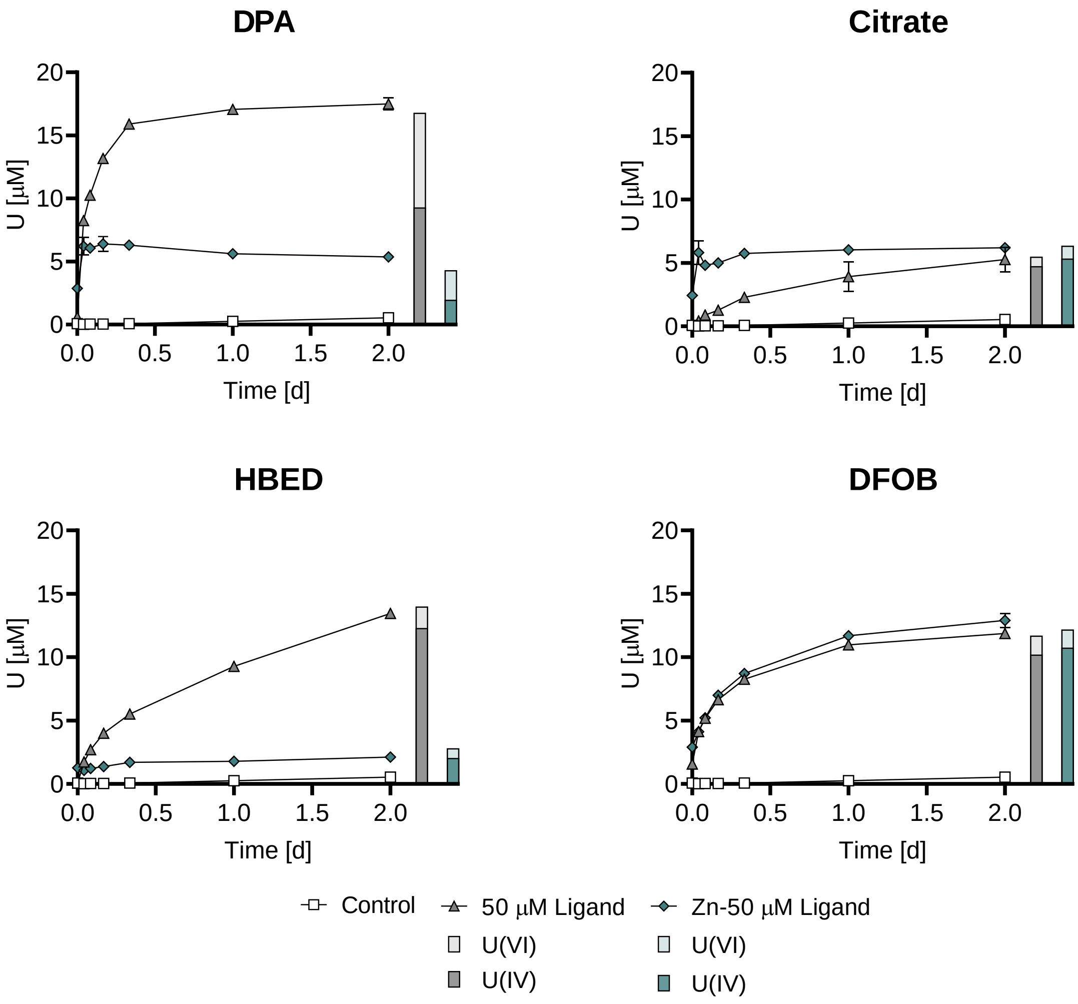

Figure S5. Effect of 50 µM Zn complexation on ligand-induced U mobilization from noncrystalline U(IV) (300 µM U) by 50 µM DPA, citrate, HBED, and DFOB at 7.0. Control treatments contained the same composition as other treatments with the exception of the ligand and competing metal. Data points show the mean (n = 2) with the ends of the error bars representing the measured values. Time t = 0 h corresponds to the moment of addition of an aliquot of noncrystalline U(IV) stock suspension to the solution containing pH buffer, electrolyte, ligand, and competing metal when applicable. Bar plots represent the redox speciation of the dissolved U as determined by ion exchange chromatography at the 2 d timepoint for each corresponding treatment and color; corresponding with Figure 1.

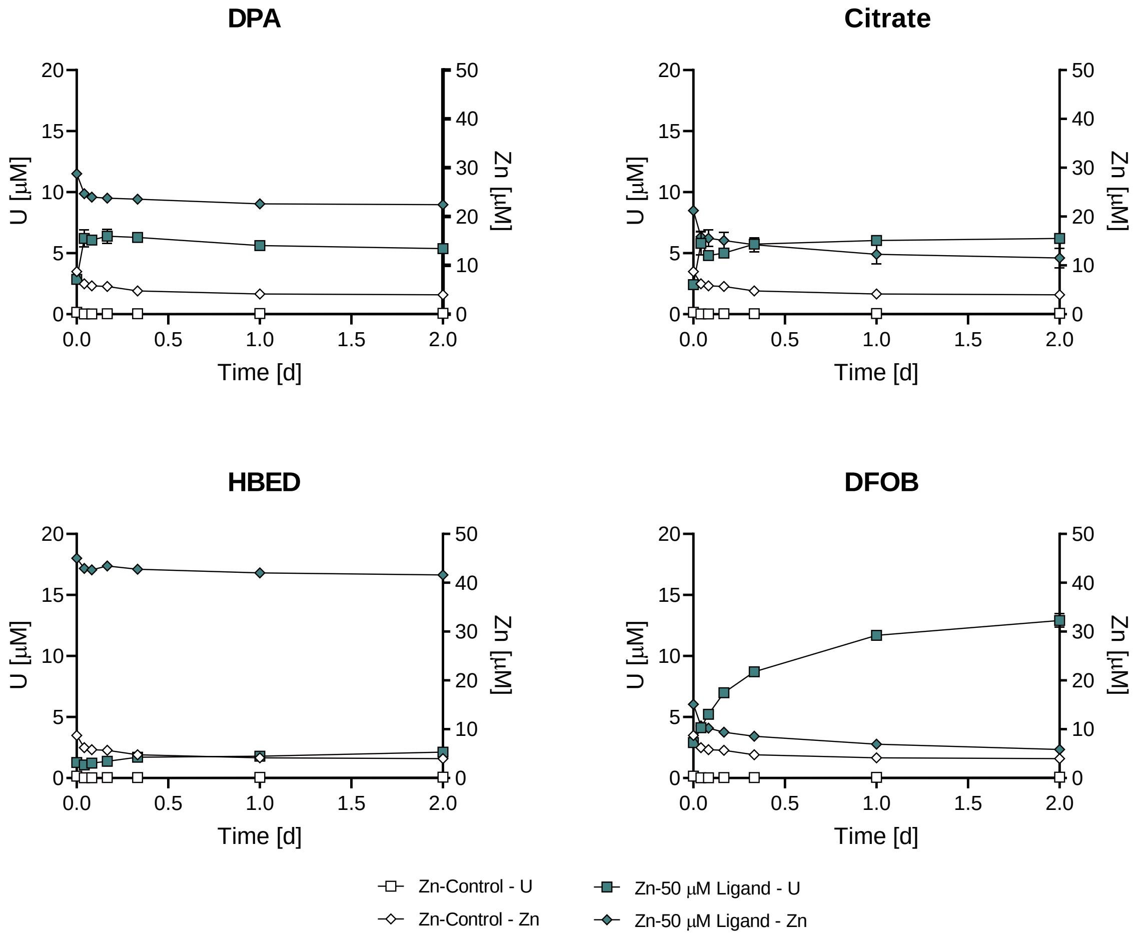

Figure S6. Dissolved U and Zn concentrations in zinc competitive displacement experiments with 50 μM DPA, citrate, HBED, and DFOB from noncrystalline U(IV) (300 μM U), at 7.0. Control treatments contained the same composition as other treatments with the exception of the ligand. Data points show the mean (n = 2) with the ends of the error bars representing the measured values. Time t = 0 corresponds to the moment of addition of an aliquot of noncrystalline U(IV) stock suspension to the solution containing pH buffer, electrolyte, ligand, and competing metal when applicable; corresponding with Figure 1.

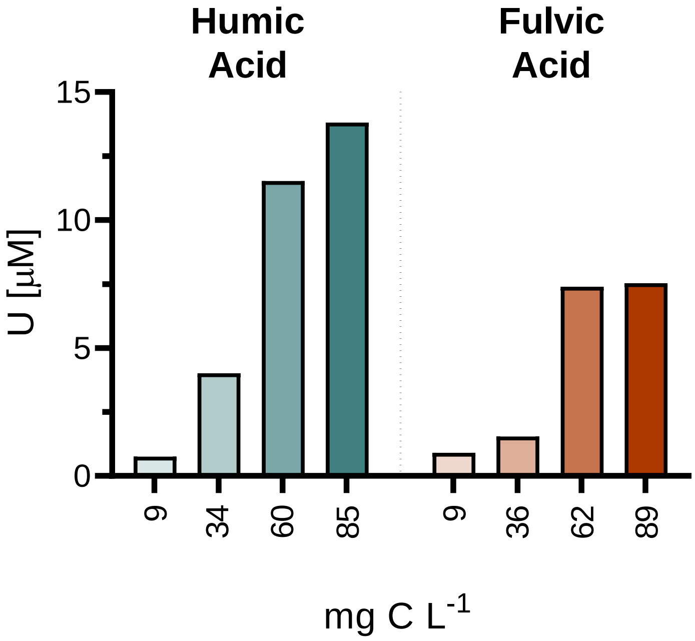

Figure S7. Summary of total U mobilized after 2 d by HA and FA from noncrystalline U(IV) (300 µM U) at concentrations of 9, 34, 60, and 85 mg C L^{-1} HA and 9, 36, 62, and 89 mg C L^{-1} FA at pH 7.0 corresponding with Figure 2.

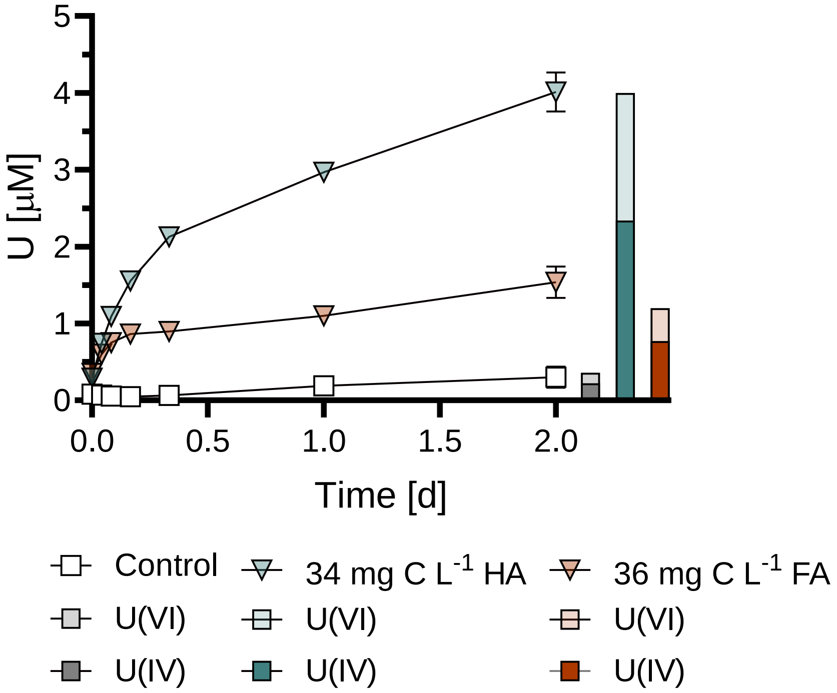

Figure S8. Humic substance-induced U mobilization from noncrystalline U(IV) (300 µM U) by 34 mg C L^{-1} HA or 36 mg C L^{-1} FA at 7.0. Control treatments contained the same composition as other treatments

with the exception of the HS. Data points show the mean (n = 2) with the ends of the error bars representing the measured values. Time t = 0 h corresponds to the moment of addition of an aliquot of noncrystalline U(IV) stock suspension to the solution containing pH buffer, electrolyte, and humic substance. Bar plots represent the redox speciation of the dissolved U as determined by ion exchange chromatography at the 2 d timepoint for each corresponding treatment and color.

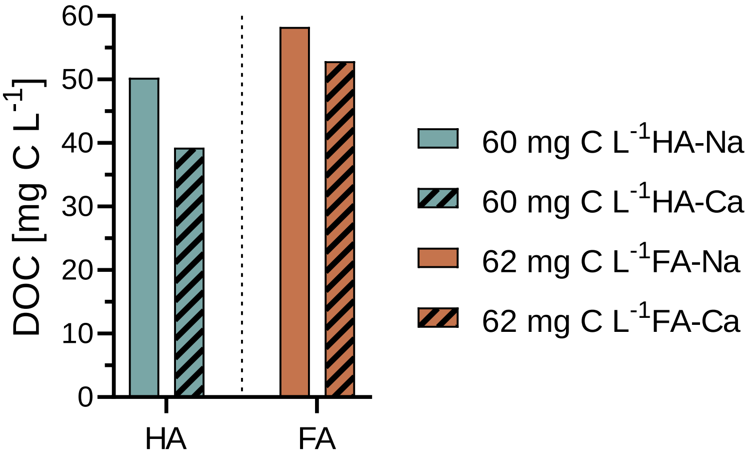

Figure S9. DOC concentrations after filtration (0.45 μm, nylon) of solutions containing 60 mg C L^{-1} HA or 62 mg C L^{-1} FA with either NaCl or $CaCl_2$ as electrolyte (I = 10 mM).

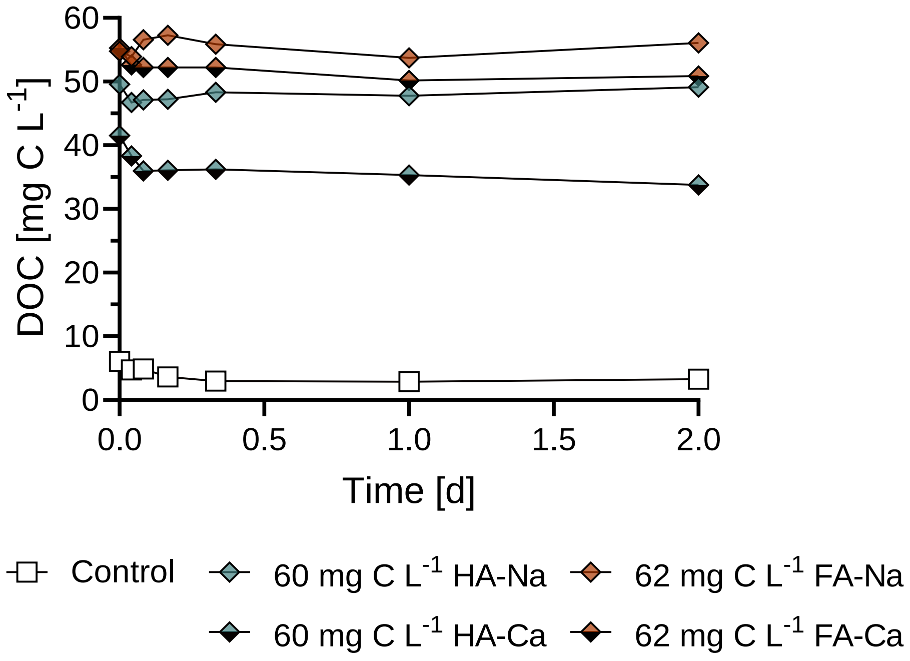

Figure S10. DOC concentrations over time from 60 mg C L^{-1} HA or 62 mg C L^{-1} FA prepared in either NaCl or $CaCl_2$ before addition to noncrystalline U(IV) (300 μM U) at pH 7.0 (± 0.3 pH units in absence of organic buffer). Electrolyte solutions of NaCl and $CaCl_2$ were prepared to account for a final ionic strength of 10 mM. Control treatments contained the same composition as other treatments with the exception of the HS. Time t = 0 h corresponds to the moment of addition of an aliquot of noncrystalline U(IV) stock suspension to the solution containing electrolyte, and humic substance.

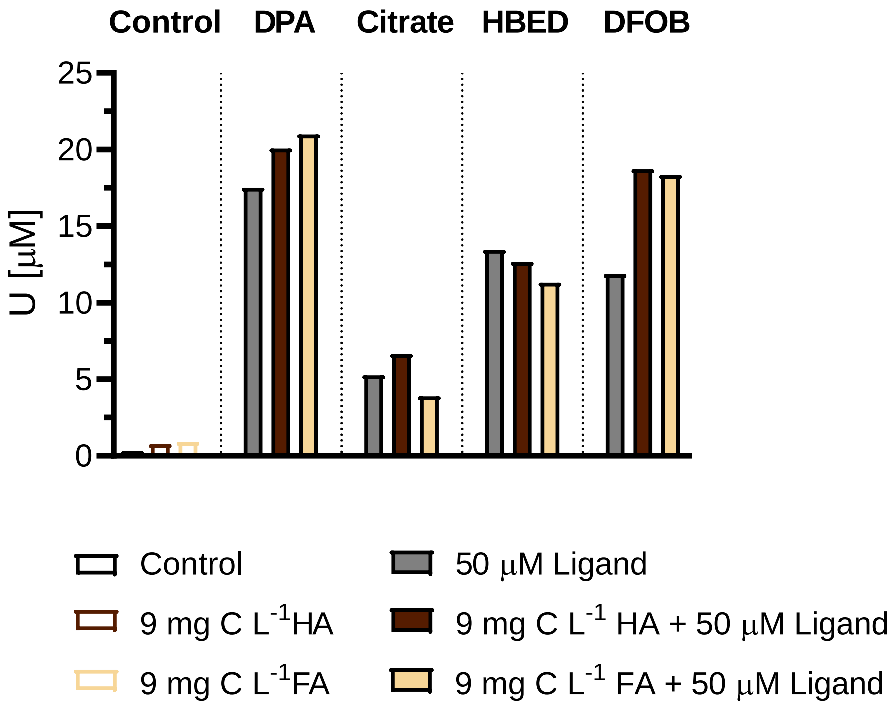

Figure S11. Summary of total U concentration mobilized throughout pre-equilibration of 9 mg C L^{-1} HA or FA with noncrystalline U(IV) (300 µM U) for 1.0 d followed by addition of 50 µM DPA, citrate, HBED, DFOB or nothing (ligand-free control) for 2 d, at pH 7.0 corresponding to Figure 4.

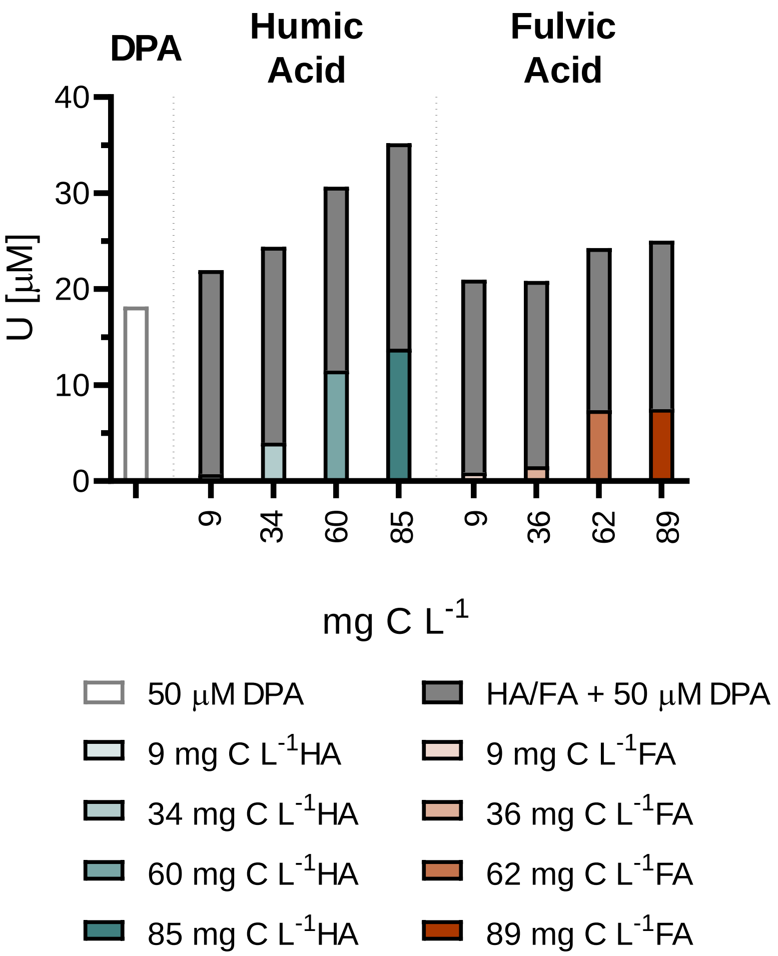

Figure S12. Summary of total U mobilized from noncrystalline U(IV) (300 µM U) by 9, 34, 60, and 85 mg C L^{-1} HA and 9, 36, 62, and 89 mg C L^{-1} FA individually (total U after 2 d) and in combination with 50 µM DPA addition (total U after 3 d including 1 d of humic substance pre-equilibration with noncrystalline U(IV)) at pH 7.0 corresponding to Figure 5.

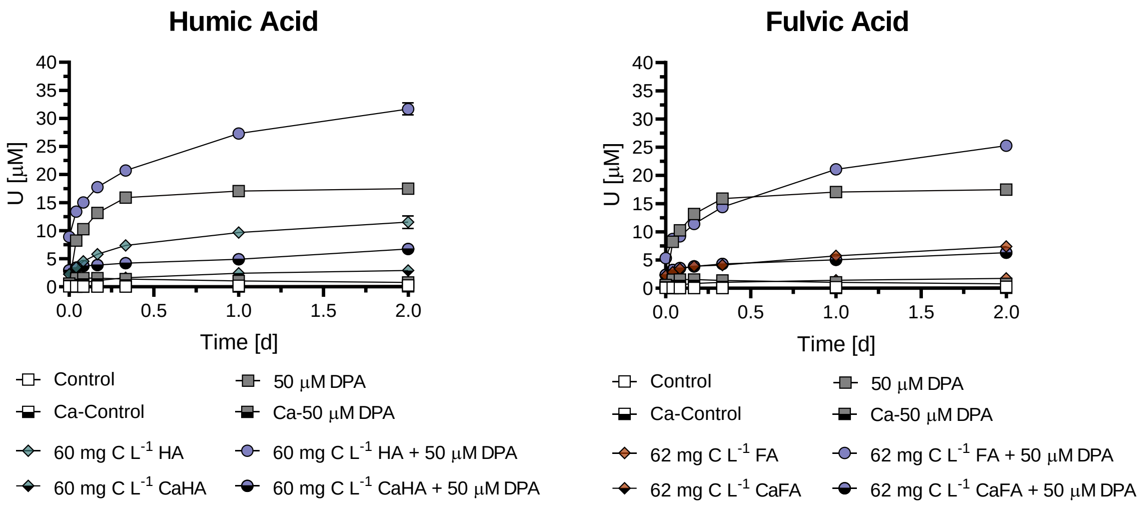

Figure S13. Overview of calcium effects (2.2 mM) on U mobilization from noncrystalline U(IV) (300 µM U) by ligand-free control, 50 µM DPA, humic substances (60 mg C L^{-1} HA, 62 mg C L^{-1} FA), and combined treatments at pH 7.0. Control treatments contained the same composition as other treatments with the exception of the ligand and HS. Control was carried out with NaCl as electrolyte while Ca-control was carried out with $CaCl_2$. Data points show the mean (n = 2) with the ends of the error bars representing the measured values.

References

1. Stoliker, D. L.; Campbell, K. M.; Fox, P. M.; Singer, D. M.; Kaviani, N.; Carey, M.; Peck, N. E.; Bargar, J. R.; Kent, D. B.; Davis, J. A., Evaluating chemical extraction techniques for the determination of uranium oxidation state in reduced aquifer sediments. *Environmental science & technology* **2013,** *47*, (16), 9225-9232.
2. Wang, X.; Johnson, T. M.; Lundstrom, C. C., Isotope fractionation during oxidation of tetravalent uranium by dissolved oxygen. *Geochimica et Cosmochimica Acta* **2015,** *150*, 160-170.
3. Smith, R.; Martell, A.; Motekaitis, R., NIST standard reference database 46. *NIST Critically Selected Stability Constants of Metal Complexes Database Ver* **2004,** *2*.
4. Bonin, L.; Cote, G.; Moisy, P., Speciation of An (IV)(Pu, Np, U and Th) in citrate media. *Radiochimica Acta* **2008,** *96*, (3), 145-152.
5. Gustafsson, J. P., Visual MINTEQ 3.0 user guide. *KTH, Department of Land and Water Recources, Stockholm, Sweden* **2011**.
6. Bombi, G. G.; Aikebaier, R.; Dean, A.; Di Marco, V. B.; Marton, D.; Tapparo, A., Complexation of 2, 6-pyridinedicarboxylic and 2, 6-pyridinediacetic acids towards aluminium (III) and iron (III). *Polyhedron* **2009,** *28*, (2), 327-335.
7. Tang, T.; Rajan, K.; Grecz, N., Mixed chelates of Ca (II)-pyridine-2, 6-dicarboxylate with some amino acids related to bacterial spores. *Biophysical journal* **1968,** *8*, (12), 1458-1474.
8. Suzuki, K.; Yamasaki, K., Stabilities of dipicolinic acid complexes with bivalent metals. *Naturwissenschaften* **1957,** *44*, (14), 396-396.
9. Motekaitis, R. J.; Martell, A. E.; Welch, M. J., Stabilities of trivalent metal complexes of phenolic ligands related to N, N'-bis (2-hydroxybenzyl) ethylenediamine-N, N'-diacetic acid (HBED). *Inorganic Chemistry* **1990,** *29*, (8), 1463-1467.
10. Eplattenier, F.; Murase, I.; Martell, A., New multidentate ligands. VI. Chelating tendencies of N, N′-Di (2-hydroxybenzyl) ethylenediamine-N, N′-diacetic acid. *Journal of the American Chemical Society* **1967,** *89*, (4), 837-843.
11. Ma, R.; Motekaitis, R. J.; Martell, A. E., Stability of metal ion complexes of N, N′-bis (2-hydroxybenzyl) ethylenediamine-N, N′-diacetic acid. *Inorganica chimica acta* **1994,** *224*, (1-2), 151-155.
12. Northover, G. H.; Mao, Y.; Blasco, S.; Vilar, R.; Garcia-España, E.; Rocco, C.; Hanif, M.; Weiss, D. J., Synergistic use of siderophores and weak organic ligands during zinc transport in the rhizosphere controlled by pH and ion strength gradients. *Scientific reports* **2022,** *12*, (1), 1-15.
